# Integrative analysis of metabolism subtypes and identification of prognostic metabolism-related genes for glioblastoma

**DOI:** 10.1042/BSR20231400

**Published:** 2024-03-22

**Authors:** Jiahui Li, Yutian Wei, Jiali Liu, Shupeng Cheng, Xia Zhang, Huaide Qiu, Jianan Li, Chuan He

**Affiliations:** 1 Department of Rehabilitation Medicine, The Affiliated Jiangsu Shengze Hospital of Nanjing Medical University, Suzhou, Jiangsu Province 215228, China; 2 Center of Rehabilitation Medicine, The First Affiliated Hospital of Nanjing Medical University, Nanjing, Jiangsu Province 210029, China; 3 Neurovascular Center, Changhai Hospital, Naval Medical University, Shanghai, 200433, China; 4 Center of Rehabilitation Medicine, Honghui Hospital, Xi'an Jiaotong University, Xi'an, Shanxi Province 710054, China; 5 Faculty of Rehabilitation Science, Nanjing Normal University of Special Education, Nanjing, Jiangsu Province 210038, China

**Keywords:** glioblastoma, metabolic networks and pathways, metabolism, prognosis

## Abstract

Increasing evidence has demonstrated that cancer cell metabolism is a critical factor in tumor development and progression; however, its role in glioblastoma (GBM) remains limited. In the present study, we classified GBM into three metabolism subtypes (MC1, MC2, and MC3) through cluster analysis of 153 GBM samples from the RNA-sequencing data of The Cancer Genome Atlas (TCGA) based on 2752 metabolism-related genes (MRGs). We further explored the prognostic value, metabolic signatures, immune infiltration, and immunotherapy sensitivity of the three metabolism subtypes. Moreover, the metabolism scoring model was established to quantify the different metabolic characteristics of the patients. Results showed that MC3, which is associated with a favorable survival outcome, had higher proportions of isocitrate dehydrogenase (IDH) mutations and lower tumor purity and proliferation. The MC1 subtype, which is associated with the worst prognosis, shows a higher number of segments and homologous recombination defects and significantly lower mRNA expression-based stemness index (mRNAsi) and epigenetic-regulation-based mRNAsi. The MC2 subtype has the highest T-cell exclusion score, indicating a high likelihood of immune escape. The results were validated using an independent dataset. Five MRGs (*ACSL1, NDUFA2, CYP1B1, SLC11A1*, and *COX6B1*) correlated with survival outcomes were identified based on metabolism-related co-expression module analysis. Laboratory-based validation tests further showed the expression of these MRGs in GBM tissues and how their expression influences cell function. The results provide a reference for developing clinical management approaches and treatments for GBM.

## Background

Glioblastoma (GBM) is the most prevalent primary malignant brain tumor type [1]. The prognosis of GBM is poor, with a median overall survival of only 15 months and a 5-year survival rate of <5% [2,3]. Conventional methods, including surgical resection, chemotherapy, and radiotherapy, are often used for GBM treatment. However, these methods have led to only limited improvements in survival time. Moreover, a few patients with GBM still experience tumor recurrence after treatment [4]. Therefore, novel drugs, immunotherapy strategies, and other GBM treatment strategies are needed [5,6].

A hallmark of cancer cells is metabolic reprogramming, manifested primarily by increased glycolytic function [7]. Dysregulation of cell metabolism plays a significant role in the development and progression of cancer through modulation of the tumor immune microenvironment [8–10]. Metabolic heterogeneity exists between tumors and within tumors [11,12] and is particularly relevant to typing, prognosis, and treatment of cancer. A comprehensive understanding of metabolic heterogeneity in cancer cells may assist in prognosis prediction and the development of novel therapeutic strategies. In recent years, advances in omics technologies have improved the prognosis and treatment of tumors. For instance, Follia et al. [13] identified four metabolic pancreatic ductal adenocarcinoma subtypes with different prognostic outcomes by analyzing transcriptomic and genomic data on the expression of glycolytic genes. Bidkhori et al. [14] conducted a metabolic network-based analysis using multiomics data and classified hepatocellular carcinoma into three subtypes to develop novel treatment strategies. However, the role of metabolic heterogeneity and relationships between changes in gene expression and dysregulation of metabolic pathways, molecular subtypes, and prognosis in GBM remain insufficiently understood.

Here, we identified three metabolism subtypes (MC1, MC2, and MC3) of GBM based on the expression of 2752 metabolism-related genes (MRGs) via clustering analysis of data obtained from The Cancer Genome Atlas (TCGA). Furthermore, we analyzed the clinical, metabolic, and immune characteristics of these metabolism subtypes and identified significant differences in clinical outcomes, genomic changes, immune infiltration, and immunotherapy response. These results may assist in improving the prediction of prognosis and developing future therapeutic strategies for GBM.

## Material and methods

### Data sources and sample collection

The RNA-sequencing data (raw counts) of GBM samples were downloaded from the TCGA database (https://www.cancer.gov/tcga). The dataset consisted of 153 primary GBM samples with clinical data. Gene expressions with >50% missing data in the samples were removed from further analysis. Two datasets of glioma samples (mRNAseq_693 and mRNAseq_325) were downloaded from the Chinese Glioma Genome Atlas (CGGA) database (http://www.cgga.org.cn/). Batch effects were removed using the combat function of the *sva* package [15]. A total of 218 primary GBM samples with clinical data were eligible for analysis. A total of 2752 MRGs were collected, reportedly encoding all known human metabolic enzymes and transporters [16].

GBM tissues and matched adjacent tissues were collected during surgery. The specimens were frozen in liquid nitrogen and stored at −80°C until immunohistochemical staining and protein extraction. Informed consent was obtained from each patient. The study was approved by the Ethics Committee of the First Affiliated Hospital of Nanjing Medical University.

### Identification of metabolism subtypes

A total of 293 out of 2752 MRGs were excluded, as these genes were not present in the TCGA-GBM dataset. The remaining 2459 genes were included for further analysis. Univariate Cox regression identified 269 survival-related genes (Cox *P*<0.05). A consensus matrix was constructed using ConsensusClusterPlus [17] based on the expression of these genes to reveal metabolism subtypes. Moreover, the *Partitioning Around Medoids (PAM)* algorithm and *canberra* as the measurement distance were used, and 1000 bootstraps were performed. Each bootstrap process included 80% of the available data in the training set. The number of clusters was set to 2-6. The optimal number of clusters was determined by constructing the consistency matrix and calculating the consistency cumulative distribution function (CDF) [17,18].

### Immune cell infiltration

The CIBERSORT algorithm (https://cibersort.stanford.edu/) was used to quantify the relative abundance of 22 immune cells in GBM. Moreover, the EPIC [19], Estimate [20,21], Xcell [22], and MCPcounter [23] algorithms were used to calculate the proportion of immune cells.

### Gene set variation analysis (GSVA) and functional annotation

GSVA was performed using the *GSVA* R package to investigate metabolic pathways of metabolism subtypes and associated biological processes. The gene set used for GSVA analysis contained 113 metabolic pathways downloaded from a previous study [24]. The *clusterProfiler* package was used for functional annotation [25].

### Determination of mutation profiles of metabolism subtypes

The differences in genomic variations between the three metabolism subtypes were further investigated in the TCGA cohort. Mutation data were obtained using the *TCGAmutation* package. Aneuploidy score, fraction altered, number of segments, tumor mutation burden, and homologous and recombination defects were analyzed in each metabolism subtype. Similarly, somatic mutations and copy number variants were analyzed and visualized using the *maftools* package.

### Tumor heterogeneity analysis of metabolism subtypes

To evaluate tumor heterogeneity among metabolism subtypes, genomic characteristics, including tumor purity, genomic ploidy, amplification, and intratumoral heterogeneity, were obtained from a previous study [26]. The stemness index, including the mRNA expression-based stemness index (mRNAsi) and epigenetic-regulation-based mRNAsi (EREG-mRNAsi) described by Malta et al. [27], was used to investigate molecular heterogeneity within tumors. A higher stemness index is associated with active biological processes in cancer stem cells and greater tumor dedifferentiation [27].

### Difference in immune infiltration and immunotherapy responses between metabolism subtypes

We used five algorithms, including EPIC, MCPcounter, ESTIMATE, CIBERSORT, and xCell, to evaluate immune cell infiltration in the TCGA dataset. TIDE (http://tide.dfci.harvard.edu/) was used to estimate the efficacy of immunotherapy in the three metabolism subtypes. Higher TIDE prediction scores indicate a higher possibility of immune escape and a lower efficacy of immunotherapy. The T-cell dysfunction score, T-cell exclusion score, and predicted immunotherapy response status of the metabolism subtypes were determined using the TCGA and CGGA datasets.

### Development of the MRG score for GBM samples

We used principal component analysis (PCA) to quantify the metabolism-related characteristics of the patients in different cohorts and develop an MRG score. MRGs with significant prognostic values were analyzed using PCA in the TCGA, and the first two components were used to calculate the metabolism index of each sample. The formula used for calculating the MRG score was as follows:

$$\text{MRG score}=\sum (\text{PC}1_{\text{i}}+\text{PC}2_{\text{i}}),$$

where* i* represents the prognostic MRG.

### Correlation of the MRG score with immune infiltration and metabolism pathways

We used the single-sample gene set enrichment analysis method to evaluate the abundance of each immune cell and investigate the correlation between the MRG score and immune cell features [28]. The abundance of 28 immune cells in the metabolic subtypes of the cohort was analyzed. The correlation between the MRG score and subtype-specific metabolic pathways was investigated.

### Identification of metabolism-related co-expression modules

Weighted gene correlation network analysis (WGCNA) was performed to identify metabolism-related co-expression modules using the *WGCNA* package [29]. Samples were filtered through good sample tests, and hierarchical clustering was applied to detect outliers further. An expression matrix was constructed by calculating the connection strength between filtered MRGs. A suitable soft-threshold power (β) was selected to ensure the constructed co-expression network conformed to the scale-free network. Next, the expression matrix was converted to an adjacency matrix, and the topological overlap matrix (TOM) was created. Based on TOM, we used the average-linkage hierarchical clustering method to cluster genes according to the mixed dynamic shear tree standards, and the number of genes in each module was at least 30. After determining the gene module by the dynamic shearing method, we calculated the eigengene value of each module, then clustered the modules. Identification of co-expression modules was performed using the following parameters: height = 0.25, deepSplit = 2, minModuleSize = 30 [30]. The distribution of the identified modules among different metabolism subtypes was calculated, and the correlations between each module and clinical traits were assessed using the gene significance (GS) and module membership (MM) analyses. Functional enrichment of these identified modules was conducted using the *clusterprofiler* R package (corrected *P*<0.05), and the top 10 biological processes in Gene Ontology were visualized.

Correlation coefficients between the modules and MRGs were calculated for the identified modules. MRGs with correlation coefficients >0.75 were selected. To identify hub MRGs, a protein–protein interaction (PPI) network was constructed using the STRING database (http://string-db.org/) [29]. Topological properties, including degree, closeness, betweenness, and eigenvector, were further analyzed, and genes with high topological properties (top 50%) were used to create a Venn diagram as these are more likely to be crucial in the network [31]. The Kaplan–Meier method was applied to select hub MRGs correlated with survival outcomes.

### Cell culture

Validation tests were performed to evaluate further hub MRGs that correlated with survival outcomes. GBM cells, including NHA, U87 (HTB-14), LN229 (CRL-2611), and T98G (CRL-1690), were purchased from the ATCC (American Type Culture Collection) and U251 (TCH-C366) was purchased from Cas9X. Dulbecco’s modified Eagle’s medium (DMEM, Gibco) with 10% fetal bovine serum (Gibco) and streptomycin/penicillin (1%, Gibco) was used to culture cells incubated with 5% CO_2_ at 37°C until 80–85% confluence.

### RT-qPCR

Following the manufacturer’s instructions, the RNA of NHA, U87, LN229, T98G, and U251 cells was extracted with the TRIzol Kit (Invitrogen), and cDNA was obtained using the HiScript III RT SuperMix for qPCR (Vazyme). Real-time PCR was performed using AceQ qPCR SYBR Green Master Mix (Vazyme), and primers were obtained from GenScript (Nanjing, China). The RT-qPCR-related primers used are listed in Supplementary Table S1.

### Plasmid and siRNA transfection and lentiviral transduction

The plasmid was designed and synthesized using Genechem software. To ensure the efficiency of inhibition of gene expression by siRNA, three different target sequences of small interfering RNAs for each gene were designed (Supplementary Table S2). As specified in the manufacturer’s protocol, the plasmid was transfected into cells using Lipofectamine 3000 (Invitrogen), and siRNAs were transfected into the cells using DharmaFECT4 (Dharmacon).

### Western blotting

The protein expression levels were determined using Western blotting. Total protein was extracted from cells and GBM tissues and matched adjacent tissues using RIPA Lysis Buffer (Beyotime), and the protein concentration was determined using the BCA assay kit (ThermoFisher Scientific). Using sodium dodecyl sulfate-polyacrylamide gel electrophoresis, equal amounts of proteins (20 μg) per sample were separated and transferred onto polyvinylidene fluoride membranes (Millipore). The blots were then incubated with primary antibodies against COX6B1 (1:1000, ab131277, Abcam), SLC11A1 (1:1000, ab211448, Abcam), ACSL1 (1:1000, ab177958, Abcam), NDUFA2 (1:500, GTX32741, GeneTex), CYP1B1 (1:500, AF301782, AiFang), E-cadherin (1:10000, ab40772, Abcam), N-cadherin (1:2000, 66219-1-Ig, Proteintech), vimentin (1:20000, 60330-1-Ig, Proteintech), Cdk4 (1:5000, ab108357, Abcam), Cdk6 (1:50000, ab124821, Abcam), Cyclin D1 (1:10000, ab134175, Abcam), and GAPDH (1:10000, 60004-1-Ig, Proteintech).

### Colony formation and CCK-8 assays

U251 or U87 cells were seeded into six-well plates (500 cells per well) and cultured in DMEM containing 10% FBS for 2 weeks. The colonies were fixed with 4% paraformaldehyde and stained with 0.1% Crystal Violet when visible to the naked eye. Colonies were photographed using a scanner (Microtek, China) and counted manually in a blind fashion. Cell proliferation was determined on transfected cells cultured in 96-well plates for 1, 2, 3, or 4 d using a CCK-8 kit (Dojindo, Japan) following the manufacturer’s instructions.

### 5-Ethynyl-2-deoxyuridine (EdU) incorporation assays

EdU incorporation assays were performed according to the manufacturer’s instructions using a KFluor 488 Click-iT EdU Imaging Kit (KeyGEN). A minimum of 50 cells were randomly selected from each field. Fluorescence intensities were calculated from five fields, and images were captured using a fluorescence microscope (Carl Zeiss, Germany).

### Immunohistochemistry

A total of 10 GBM tissues and matched adjacent tissues were used for immunohistochemical staining. Briefly, tissues were fixed in 4% paraformaldehyde (Servicebio, Wuhan, China) for 24 h before routine decalcification and dehydration through an ethanol gradient. The tissues were cut into 4 µm sections using a rotary microtome. After deparaffinization and rehydration, sections were immersed in boiling citrate buffer for 30 min. After blocking with BSA for 1 h, the sections were incubated with the appropriate primary antibodies overnight at 4°C. The primary antibodies used were COX6B1 (1:80, ab131277; Abcam), SLC11A1 (1:50, ab211448; Abcam), ACSL1 (1:100, ab177958; Abcam), NDUFA2 (1:50, GTX32741; GeneTex), and CYP1B1 (1:50, AF301782, AiFang). The sections were washed and incubated with a secondary antibody (100 μL, AFIHC001, AiFang). Finally, the sections were stained with 3,3′-diaminobenzidine and counterstained with hematoxylin before observation under a microscope.

### Statistical analysis

Statistical analyses were performed using R software (version 4.1.2) and GraphPad Prism (version 8.3.0). One-way analysis of variance (ANOVA) and Kruskal–Wallis tests were used to compare the three groups. Fisher’s test analyzed the relationship between gene mutations and metabolism subtypes. Correlation analyses were performed using Spearman’s correlation test. The Kaplan–Meier method was used for survival analysis, and the log-rank test was used for comparison. A *P*<0.05 was considered to indicate a statistically significant difference.

## Results

### Identification of metabolism subtypes

**Figure 1:**
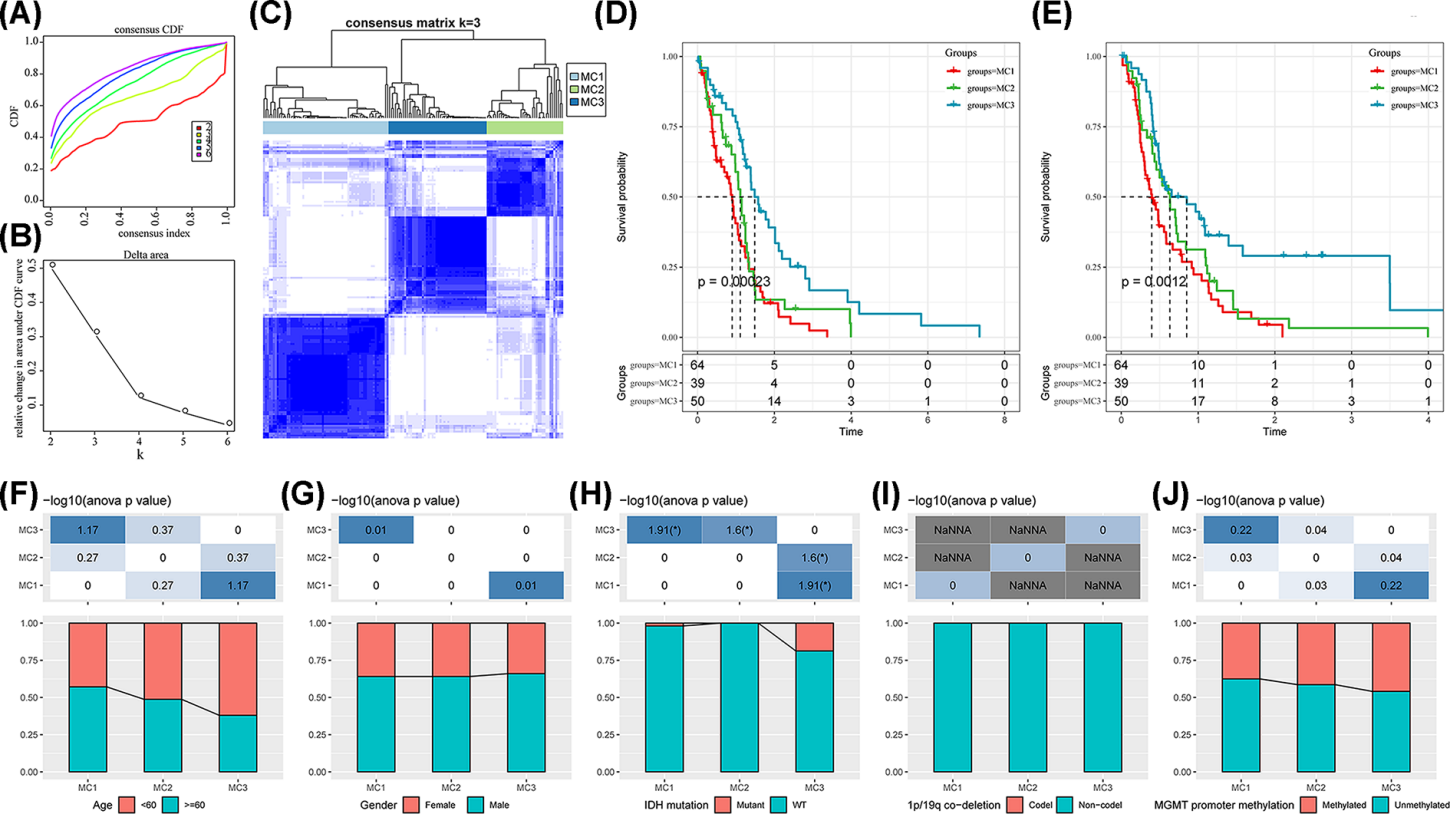
Metabolism subtypes of GBM (**A**) CDF curve based on the TCGA-GBM cohort data. (**B**) CDF delta area curve based on the TCGA-GBM cohort data. The delta area curve of consensus clustering indicated the relative change in the area under the CDF curve for each category number *k* compared with *k* – 1. The horizontal axis represents the category number k, and the vertical axis represents the relative change in the area under the CDF curve. (**C**) Clustering heatmap based on the TCGA-GBM sample data with consensus *k* = 3. (**D**) Kaplan–Meier curve for overall survival among subtypes in the TCGA cohort. (**E**) Kaplan–Meier curve for progression-free survival among subtypes in the TCGA cohort. (**F**) Age distribution of metabolism subtypes in the TCGA cohort. (**G**) Sex distribution of metabolism subtypes in the TCGA cohort. (**H**) IDH mutation status distribution of metabolism subtypes in the TCGA cohort. (**I**) 1p/19q codeletion status distribution of metabolism subtypes in the TCGA cohort. (**J**) MGMT promoter methylation status distribution of metabolism subtypes in the TCGA cohort. *P<0.05; CDF, cumulative distribution function; GBM, glioblastoma; IDH, isocitrate dehydrogenase; MGMT, O6-methylguanine-DNA methyltransferase; TCGA, The Cancer Genome Atlas.

GBM expression profiles were extracted from the RNA-sequencing data from the TCGA, and 269 metabolic genes with significant prognostic values were obtained for subsequent analysis. The three clusters demonstrated relatively stable clustering results (Figure 1A,B). Therefore, we chose the number of clusters (*k*) as three to obtain three metabolism-related clusters (MC) (Figure 1C), indicating that GBM samples can be categorized into three distinct metabolism subtypes. These three metabolism subtypes demonstrated significant prognostic differences (Figure 1D,E). The MC3 and MC1 subtypes showed favorable and poor prognoses, respectively. In addition, 269 MRGs were used to validate the metabolism subtypes in the CGGA dataset. Similarly, the prognoses of these three metabolism subtypes were significantly different in the CGGA (Supplementary Figure S1). The MC1 subtype had the worst prognosis. In contrast, a favorable prognosis was associated with MC3 compared with the other subtypes. Overall, these results suggest that the identified metabolism subtypes based on the expression profiles of 269 MRGs could potentially serve as valuable prognostic indicators for GBM patients. In order to better elucidate the significance of these three metabolism subtypes and their relevance to clinical strategies or treatments for GBM patients, the study conducted a comprehensive exploration of these three metabolism subtypes from multiple perspectives.

### Clinical characteristics of metabolism subtypes

We performed a comparative evaluation of clinical characteristics of metabolism subtypes as observed in the TCGA-GBM dataset, and no significant differences between metabolism subtypes in terms of age, sex, 1p/19q codeletion, or O-6-methylguanine-DNA methyltransferase (MGMT) promoter methylation were observed (Figure. 1F,G,I,J). The proportion of isocitrate dehydrogenase (IDH) mutants was higher in MC3 than in the other subtypes, which may explain the favorable survival outcome with this subtype (Figure 1H). The correlation between metabolism subtypes and clinical characteristics in the CGGA is shown in Supplementary Figure S1.

### Correlation of metabolism subtypes with metabolic processes

**Figure 2:**
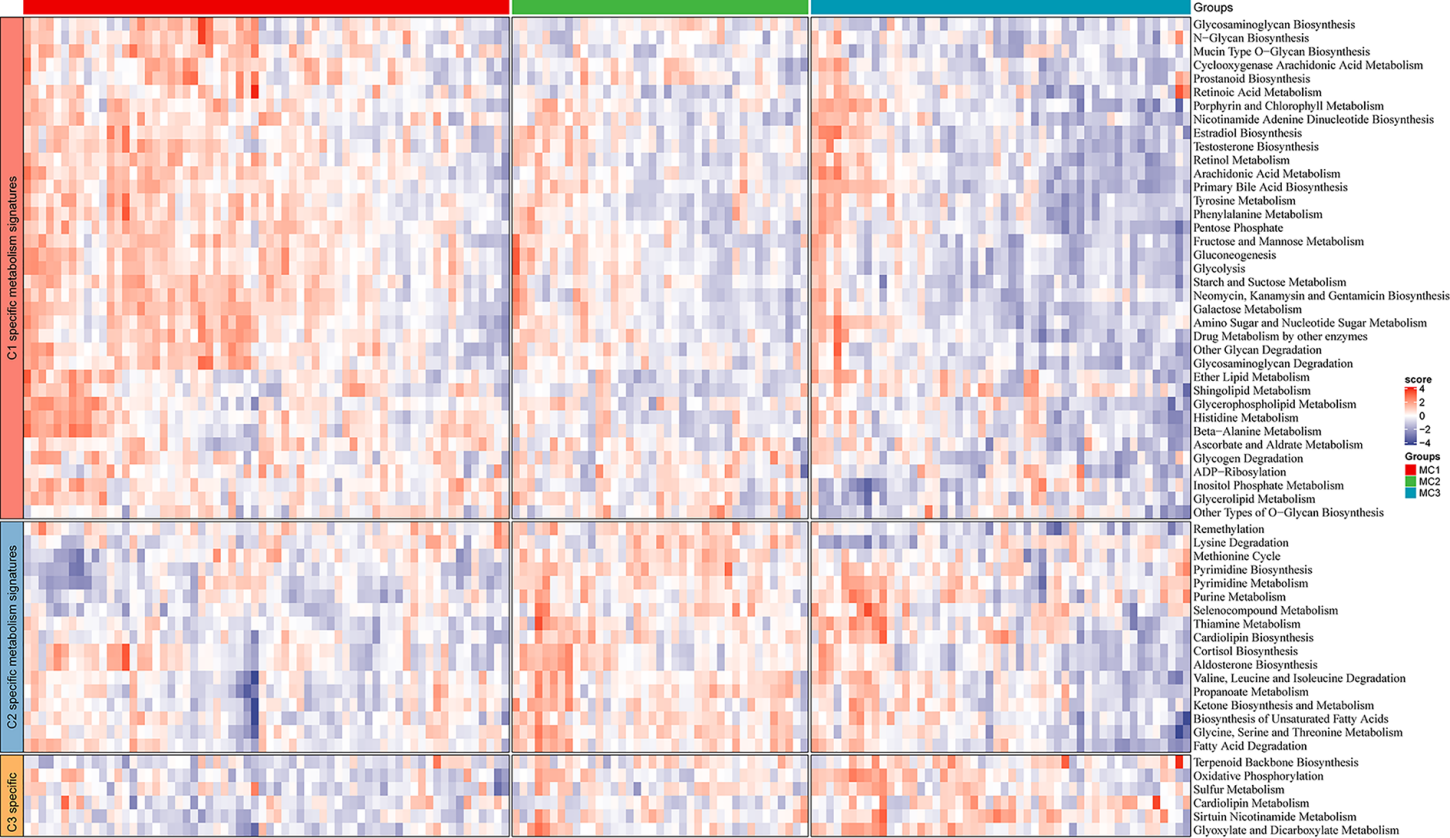
Metabolism signatures corresponding to subtypes

Since metabolism subtypes of GBM were identified based on the results of a consistent clustering analysis, we further analyzed metabolic processes in these subtypes. The *GSVA* package was used to quantify 113 metabolic processes, and differential analysis was performed to identify subtype-specific metabolic characteristics, defined as characteristics with high GSVA scores in the corresponding subtypes. The results revealed 37, 17, and 6 specific metabolic characteristics in the MC1, MC2, and MC3 subtypes, respectively (Figure 2). The MC1 subtype exhibits the most specific metabolic characteristics in comparison to the other two subtypes, suggesting its classification as a metabolically active subtype, potentially contributing to a poorer prognosis [32]. This analysis of subtype-specific metabolic characteristics sheds light on the heterogeneity of GBM metabolism and has important implications for the development of targeted therapies and personalized treatment strategies. The distinct metabolic profiles observed in each subtype could potentially serve as biomarkers for patient stratification and may guide the selection of suitable therapeutic interventions.

### Mutation profiles and heterogeneity analysis

**Figure 3:**
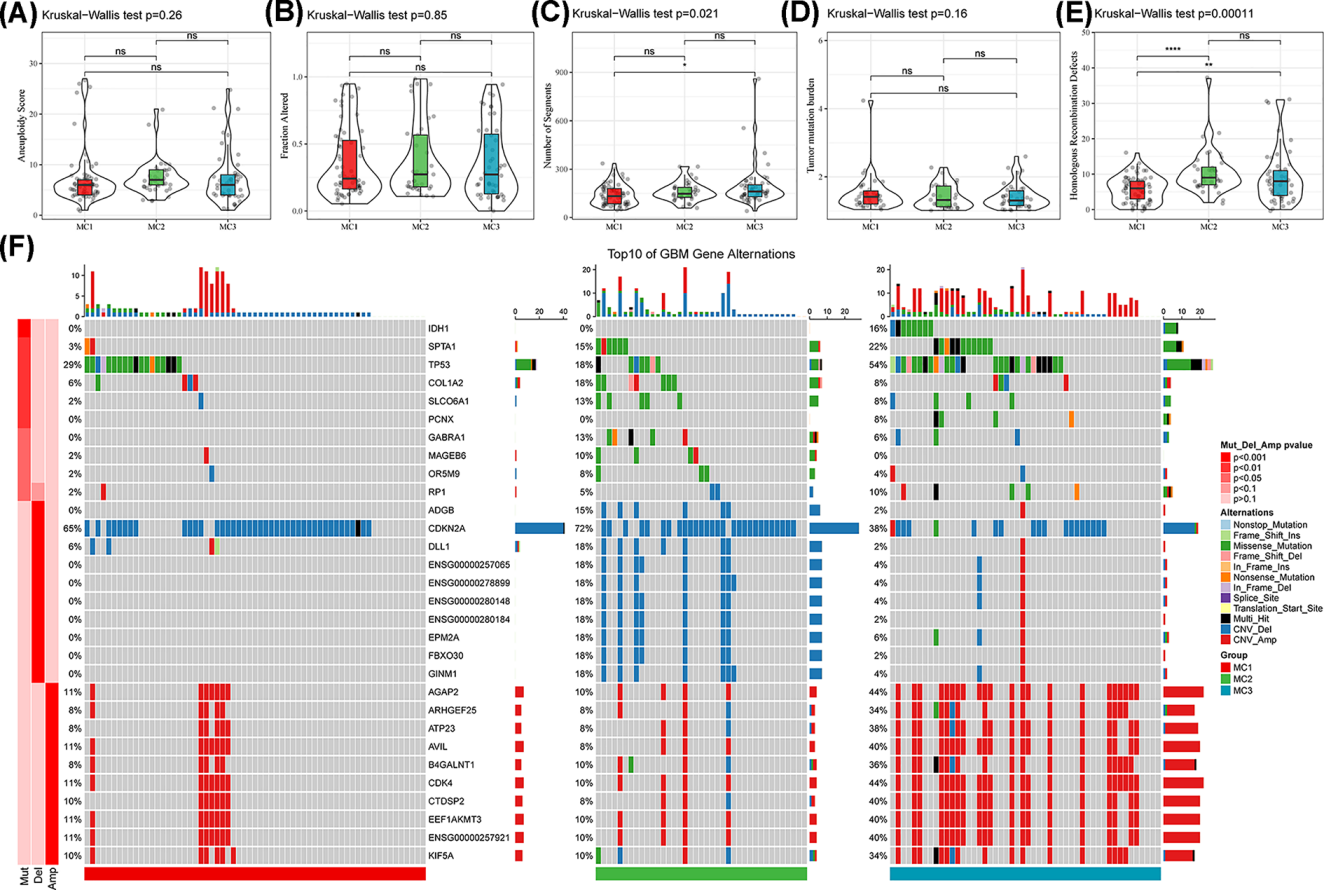
Genomic changes of metabolism subtypes in the TCGA-GBM cohort Aneuploidy score (**A**), fraction altered (**B**), number of segments (**C**), tumor mutation burden (**D**), and homologous recombination defects (**E**) were analyzed. (**F**) Analysis of somatic mutations and copy number variants in metabolism subtypes using Fisher’s test; *P<0.05, **P<0.01, ***P<0.001, ****P<0.0001; TCGA-GBM, The Cancer Genome Atlas-Glioblastoma.

The differences in genomic variations between the identified metabolism subtypes in the TCGA cohort were analyzed (Figure 3A–E). The MC1 subtype exhibited a high number of segments and homologous recombination defects. This observation suggests that the MC1 subtype may harbor unique genomic alterations that potentially contribute to its distinct metabolic characteristics and functional implications. The correlation between gene mutations and metabolism subtypes was analyzed (Figure 3F). Specifically, the MC3 subtype exhibited a notable frequency of IDH mutations, whereas no IDH mutations were observed in the MC1 or MC2 subtypes, aligning with the superior prognosis associated with the MC3 subtype.

**Figure 4:**
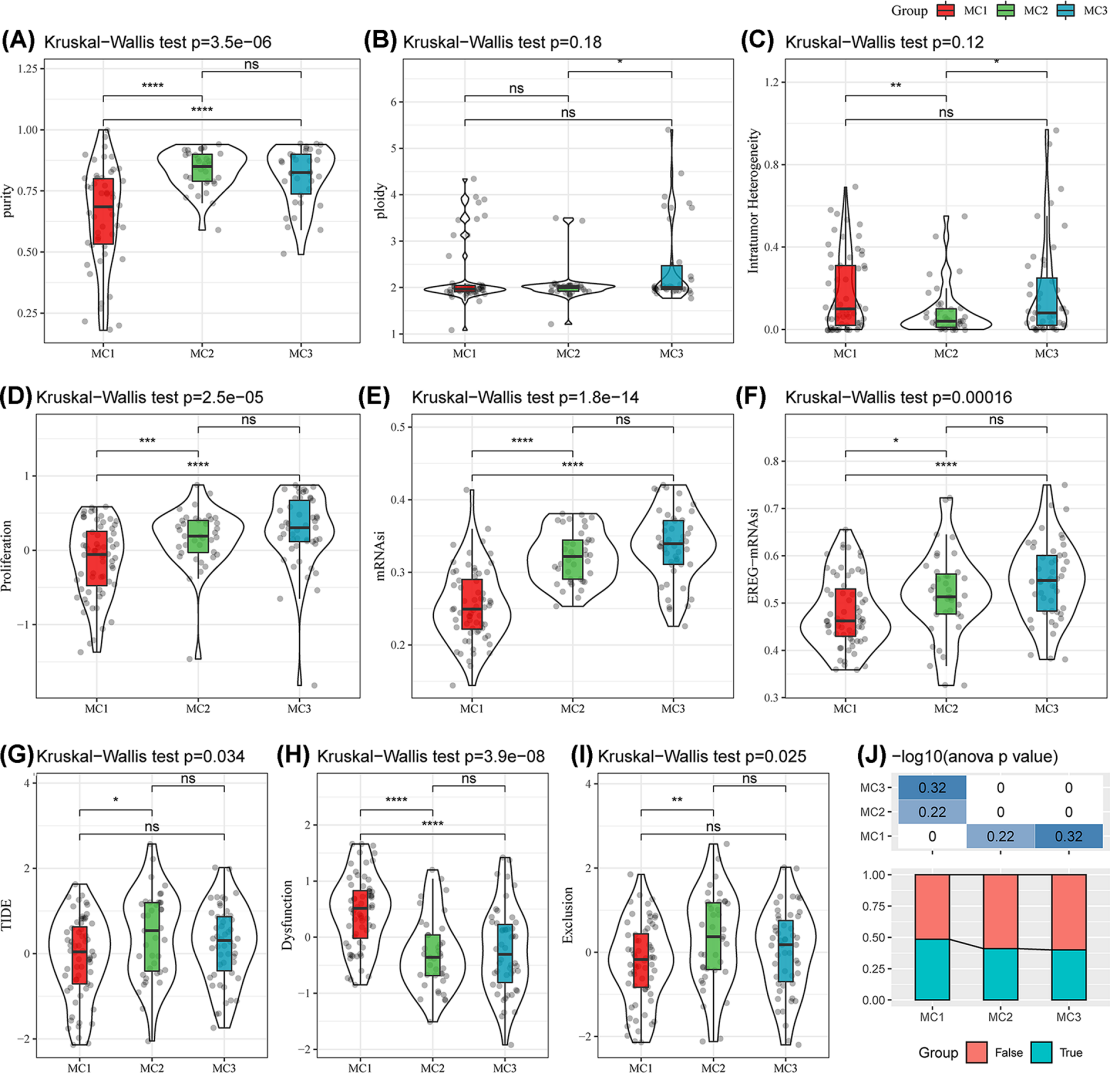
Heterogeneity and immunotherapy response across metabolism subtypes (**A**) Analysis of purity in metabolism subtypes. (**B**) Analysis of ploidy in metabolism subtypes. (**C**) Analysis of intratumor heterogeneity in metabolism subtypes. (**D**) Analysis of proliferation in metabolism subtypes. (**E**) Analysis of mRNAsi in metabolism subtypes. (**F**) Analysis of EREG-mRNAsi in metabolism subtypes. (**G**) TIDE scores of metabolism subtypes in the TCGA cohort. (**H**) T-cell dysfunction scores of metabolism subtypes in the TCGA cohort. (**I**) T-cell exclusion scores of metabolism subtypes in the TCGA cohort. (**J**) Predicted immunotherapy response status of metabolism subtypes in the TCGA cohort. *P<0.05; **P<0.01; ***P<0.001; ****P<0.0001. EREG-mRNAsi, epigenetic-regulation-based mRNA expression-based stemness index; TCGA, The Cancer Genome Atlas.

Tumor purity in the MC1 subtype was significantly lower than those in the MC2 or MC3 subtypes (Figure 4A), indicating a more complex tumor microenvironment of MC1. The proliferation of tumors in the MC1 subtype was significantly lower compared with the other two subtypes (Figure 4D). This observation implies that the MC1 subtype may possess distinct biological characteristic. The mRNAsi and EREG-mRNAsi of the MC1 subtype were significantly lower than those of the MC2 or MC3 subtypes (Figure 4E,F). The gradual loss of cell differentiation and acquisition of stem cell-like characteristics are the main factors driving tumor progression [33] and, thus, worse survival outcomes in the MC1 subtype. A high mRNAsi in GBM indicates a favorable survival outcome [34].

### Immune infiltration and immunotherapy responses in metabolism subtypes

The results of immune cell infiltration are shown in Supplementary Figure S2. The EPIC method evaluated the proportions of eight types of immune cells and the proportion of CD4^+^ T cells in the MC3 subtype was significantly higher than in the other subtypes. Among the 10 immune cell types evaluated by the MCPcounter software, the proportions of all the immune cell types except for CD8^+^ T cells and endothelial cells were significant among metabolism subtypes. The results obtained using CIBERSORT revealed no significant differences in most immune cells between the metabolism subtypes. Of the 64 immune cells evaluated by xCell, a few were significantly different between the subtypes. The evaluation results of ESTIMATE showed that the MC1 subtype had the highest immune, stromal, and ESTIMATE scores. The heatmap showing the evaluation conducted by the five immune infiltration algorithms in the TCGA-GBM cohort is provided in Supplementary Figure S3.

We analyzed the differences in immune therapy among different metabolic molecular subtypes. The results showed that MC2 had the highest TIDE score in the TCGA cohort, followed by MC3 and MC1 (Figure 4G). This suggests that patients with the MC2 subtype are less likely to benefit from immunotherapy. Moreover, we compared the predicted T cell dysfunction scores and T-cell rejection scores between the metabolism subtypes in the TCGA cohort. The T-cell dysfunction score was higher for MC1 than that of the other subtypes (Figure 4H). This may explain the poor prognosis of the MC1 subtype, despite having the highest immune infiltration. The MC2 subtype showed the highest T-cell exclusion score in the TCGA cohort (Figure 4I). Similar scores across subtypes were observed in the CGGA cohort (Supplementary Figure S4). We analyzed the predicted immunotherapy response status of the metabolism subtypes in the TCGA cohort. The response to immunotherapy was slightly higher in patients with MC1 compared with MC2 and MC3, yet no significant difference was observed (Figure 4J).

### Construction of the MRG score

**Figure 5:**
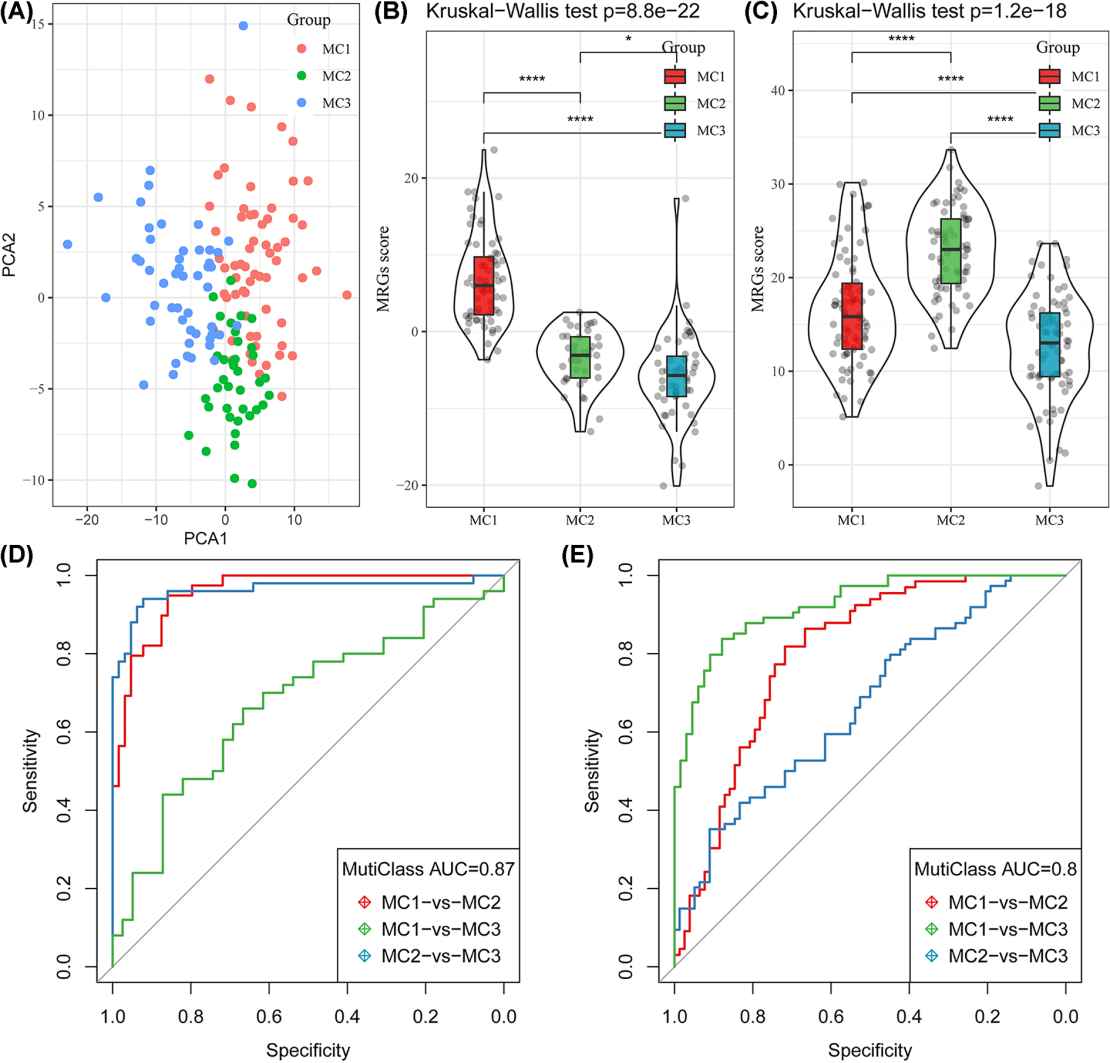
Construction of the MRG score (**A**) PCA clustering of the TCGA samples with different metabolism subtypes. (**B**) The difference in the MRG score between subtypes in the TCGA dataset. (**C**) The difference in the metabolism subtype feature scores between subtypes in the CGGA dataset. (**D**) ROC curve for the MRG score in the TCGA dataset. (**E**) ROC curve for the MRG score in the CGGA dataset. CGGA, Chinese Glioma Genome Atlas; MRG, metabolism-related gene; PCA, principal component analysis; ROC, receiver operating characteristics; TCGA, The Cancer Genome Atlas.

Samples of different subtypes could be clearly distinguished according to the first two features of the metabolism-scoring model (Figure 5A). We calculated the MRG score for each patient in the TCGA dataset based on the metabolism-scoring model. The MRG scores of metabolism subtypes were significantly different from each other (Figure 5B). The classification performance of the MRG score for the subtypes based on the ROC curve is shown in Figure 5D. Accordingly, the multiclass AUC was 0.87. We obtained results similar to those obtained from the TCGA dataset by using the same equation for the CGGA dataset. The MRG scores of different subtypes were distinct from each other (Figure 5C). The ROC curve indicated an AUC of 0.8 (Figure 5E). These results indicate that the MRG score can be used to measure the patients’ metabolic characteristics.

### Correlation of the MRG score with immune infiltration and metabolic processes

**Figure 6:**
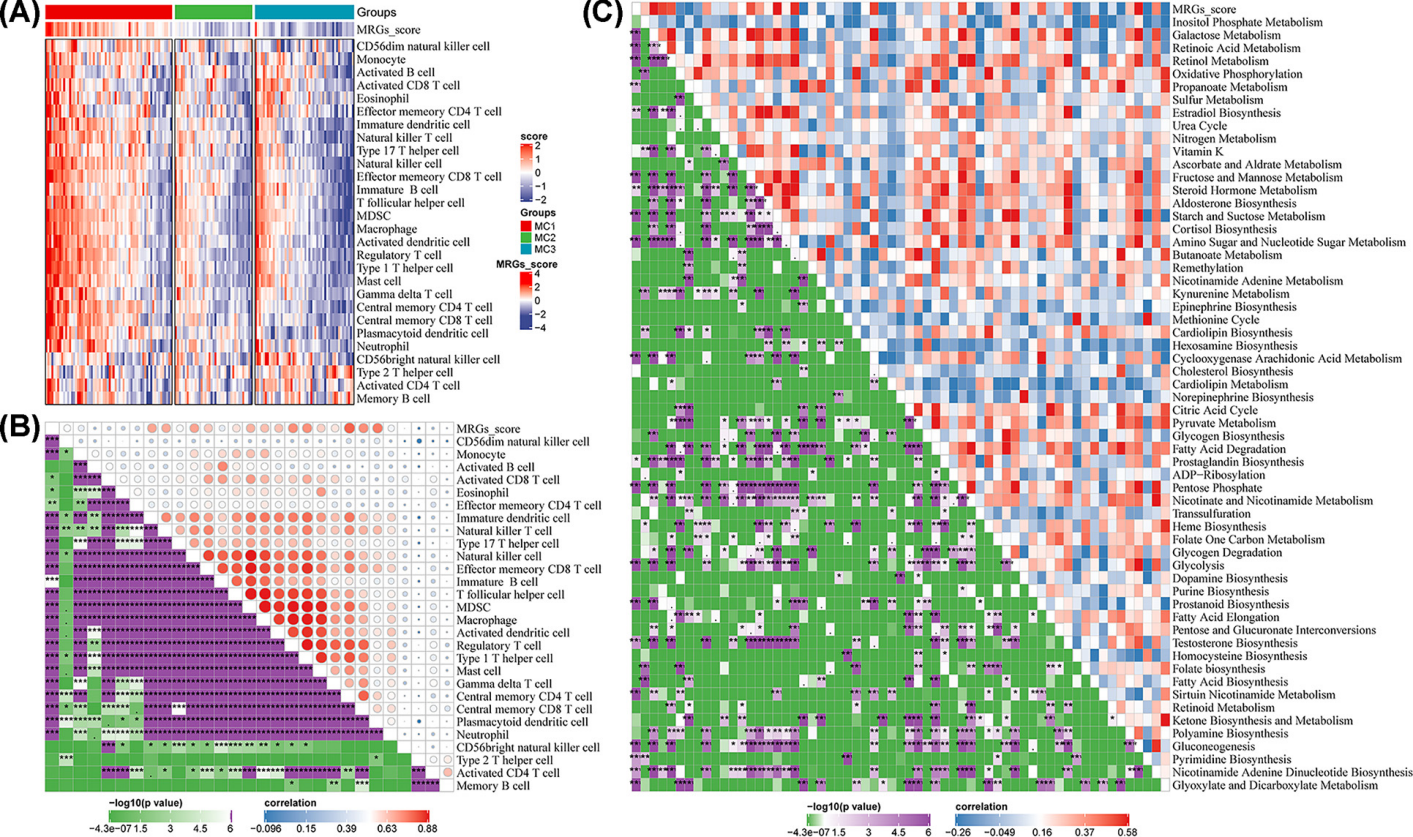
Correlation of the MRG score with immune infiltration and metabolic processes (**A**) The abundance of 28 immune cells in metabolism subtypes in the TCGA cohort. (**B**) Correlation of MRG score and abundance of 28 immune cells in the TCGA cohort. (**C**) Correlation of MRG score and multiple metabolic pathways. CGGA, Chinese Glioma Genome Atlas; MRG, metabolism-related gene; TCGA, The Cancer Genome Atlas.

The enrichment scores of most immune cells in MC1 were higher than those in the other metabolism subtypes (Figure 6A). Thus, we analyzed the relationship between the metabolic subtype feature scores in the TCGA cohort and the 28 immune cell scores and calculated the correlation between the MRG score and immune cells using Pearson correlation analysis. The subtype feature score was significantly correlated with most immune cell types (Figure 6B). For example, the metabolism subtype feature score showed a significantly positive correlation with immature dendritic cells, NK T cells, central memory CD4^+^ T cells, central memory CD8^+^ T cells, and plasmacytoid dendritic cells. Negative correlations were observed for CD56dim NK cells, monocytes, and type 17 T-helper cells. We found that MRG scores and subtype-specific metabolic pathways were closely correlated (Figure 6C). The MRG score was positively correlated with metabolic pathways, such as those of galactose metabolism, retinoic acid metabolism, and retinol metabolism, and negatively correlated with pathways of pyrimidine biosynthesis, glyoxylate, and dicarboxylate metabolism. These findings reveal the complex interaction between metabolic and immune signatures in distinct subtypes, which could yield valuable information about the underlying mechanisms influencing the tumor microenvironment.

### Identification of metabolism-related co-expression modules

**Figure 7:**
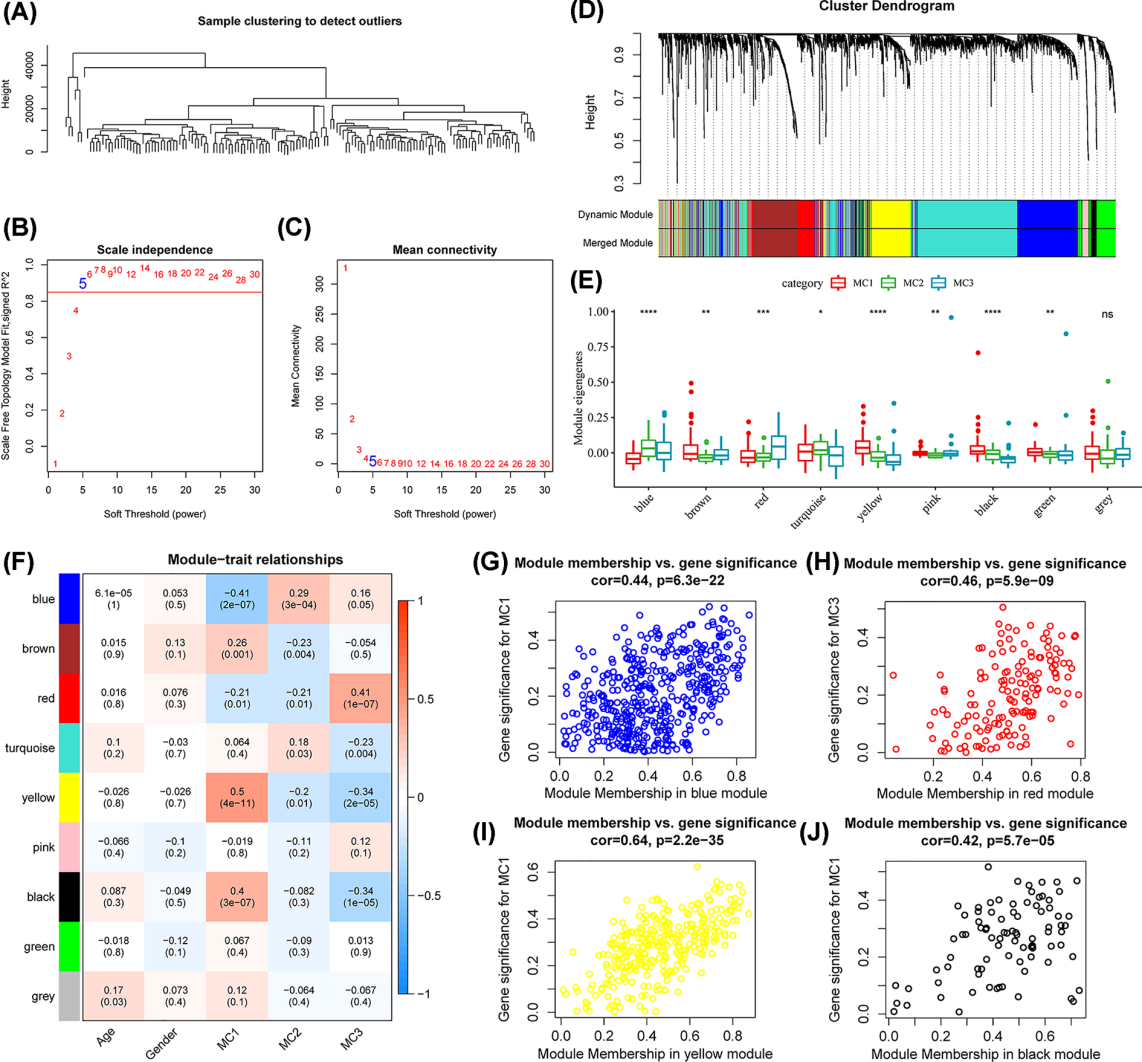
Identification of the metabolism-related co-expression module (**A**) The sample clustering tree detected no apparent outliers. (**B**) Analysis of the scale-free fit index for various soft-thresholding powers (β). (**C**) Analysis of the mean connectivity for various soft-thresholding powers. (**D**) Dendrogram of all MRGs clustered based on a dissimilarity measure (1-TOM). (**E**) Profile of module eigengenes in metabolism subtypes. (**F**) Correlations of modules and clinical traits. (**G**) Scatter diagram of module membership versus gene significance for MC1 in the blue module. (**H**) Scatter diagram for module membership vs. gene significance for MC3 in the red module. (**I**) Scatter diagram for module membership versus gene significance for MC1 in the yellow module. (**J**) Scatter diagram for module membership versus gene significance for MC1 in the black module.

The result of hierarchical clustering is shown in Figure 7A. The soft-threshold power (β) was set at 5 to ensure the constructed co-expression network conformed to the scale-free network (Figure 7B,C). The cluster dendrogram highlighted a total of nine modules. The gray module contained genes that could not be classified into any other module (Figure 7D). Subsequently, the eigengenes were compared across the metabolism subtypes (Figure 7E). Significant differences between the subtypes in eight modules (except for the gray module) were observed. In the yellow and black modules, the MC1 subtype showed higher eigengenes than the other subtypes. In contrast, the MC3 subtype exhibited the highest eigengene expression in the blue and red modules. Module-trait relationships were explored using correlation analysis. Accordingly, the MC1 subtype was positively and negatively correlated with the yellow and black modules and the blue module, respectively. The MC3 subtype was positively correlated with the red module (Figure 7F). The correlations between MM and GS for the above modules are shown in Figure 7G–J. This comprehensive analysis offers valuable insights into the intricate interactions between metabolism subtypes and associated gene modules, laying the groundwork for potential predictive biomarkers.

### Functional enrichment for the MRG co-expression module

**Figure 8:**
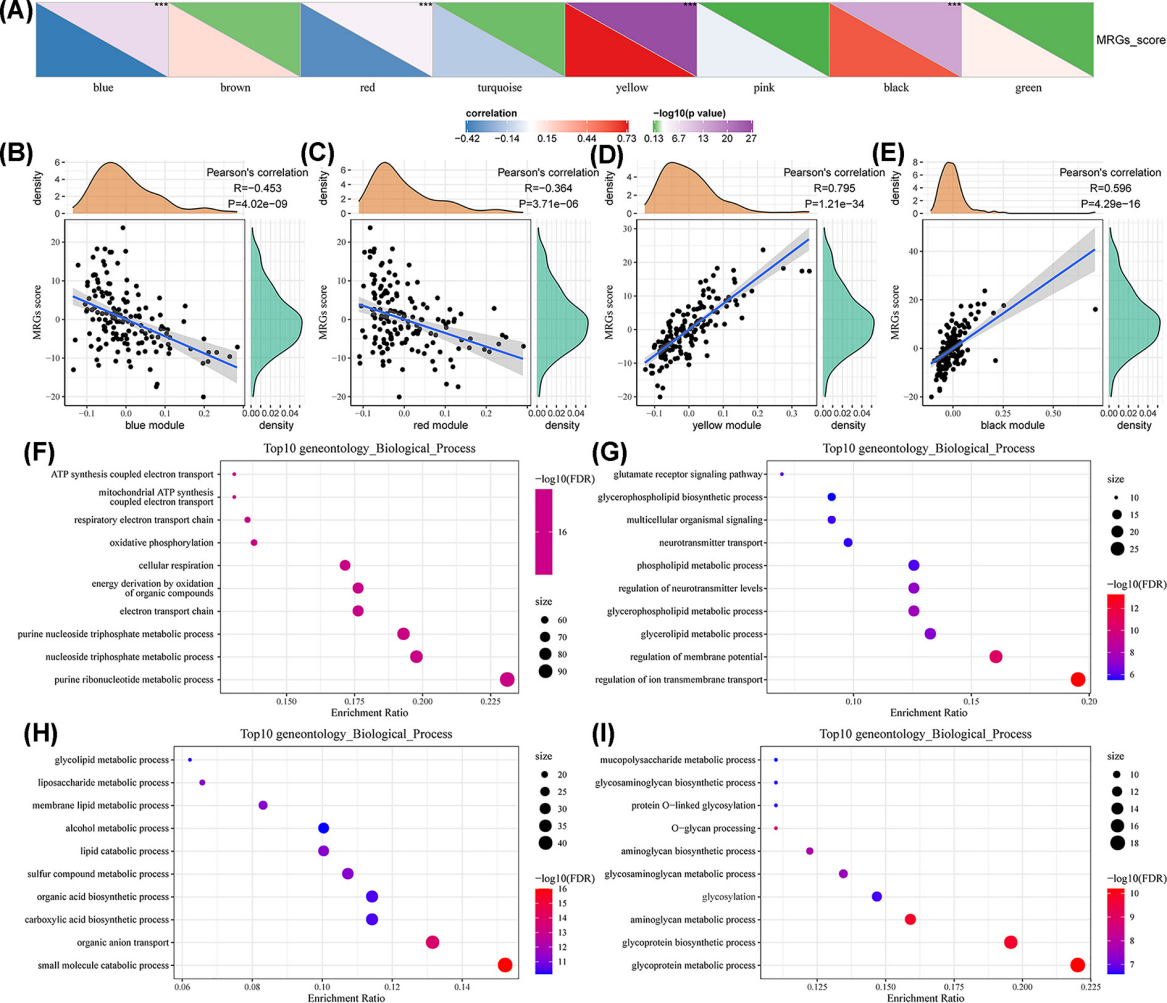
Correlation analysis of module eigengenes and enrichment analysis of modules (**A**) The overall correlation between the MRG score and module eigengenes. (**B**) Correlation between the MRG score and blue module eigengenes. (**C**) Correlation between the MRG score and red module eigengenes. (**D**) Correlation between the MRG score and yellow module eigengenes. (**E**) Correlation between the MRG score and black module eigengenes. (**F**) Enrichment analysis of the MRGs in the blue module. (**G**) Enrichment analysis of the MRGs in the red module. (**H**) Enrichment analysis of the MRGs in the yellow module. (**I**) Enrichment analysis of the MRGs in the black module. MRG, metabolism-related gene.

After excluding the gray module, we next analyzed the correlation between the MRG score and module eigengenes (Figure 8A). The eigengenes in the blue and red modules were negatively correlated with the MRG score (Figure 8B,C), whereas the eigengenes in the yellow and black modules were positively correlated (Figure 8D,E). The aforementioned four modules were selected for enrichment analysis. The results indicated that the MRGs in the blue module were enriched in genes associated with ATP synthesis-coupled electron transport, mitochondrial ATP synthesis-coupled electron transport, the respiratory electron transport chain, and oxidative phosphorylation (Figure 8F). MRGs in the red module were enriched in genes associated with glutamate receptor signaling, glycerophospholipid biosynthetic, and glycerolipid metabolic processes (Figure 8G). The enrichment results for the yellow and black modules are shown in Figure 8H,I, respectively.

**Figure 9:**
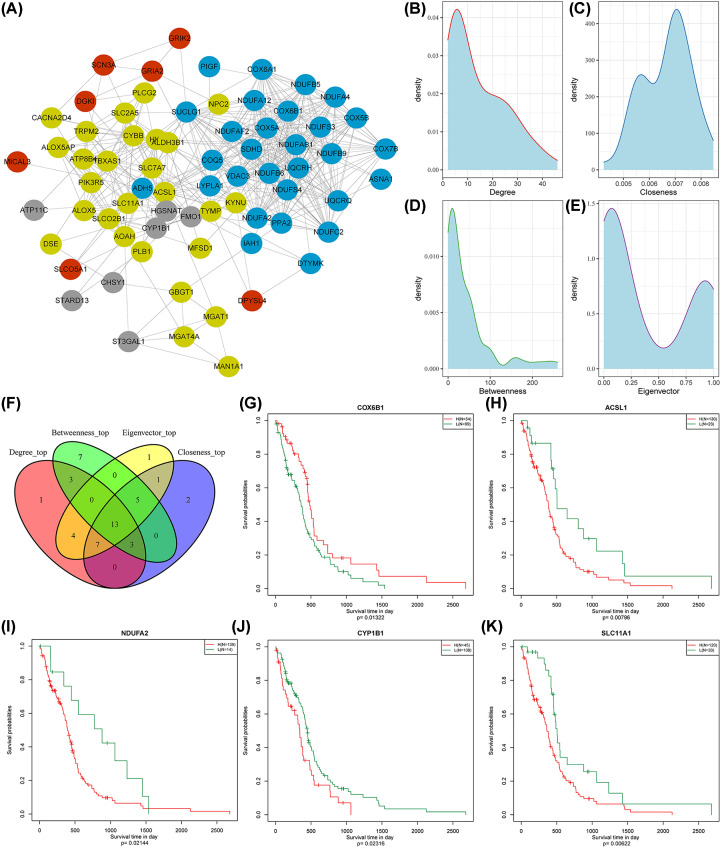
Identification of MRGs (**A**) PPI network of the key genes in the modules. The color of the nodes represents different modules. (**B**) Degree of the network. (**C**) Closeness of the network. (**D**) Betweenness of the network. (**E**) Eigenvector of the network. (**F**) Venn diagram of the top-half gene intersection. Kaplan-Meier survival curves of (**G**) *COX6B1*, (**H**) *ACSL1*, (**I**) *NDUFA2*, (**J**) *CYP1B1*, and (**K**) *SLC11A1*. MRG, metabolism-related gene; PPI, protein–protein interaction.

We selected 71 MRGs with a correlation coefficient above 0.75 in the four modules. A PPI was observed in 70 of them based on data obtained from the STRING database (Figure 9A). The interaction confidence score of the PPI network is shown in Supplementary Table S3. Topological properties were further analyzed, including degree, closeness, betweenness, and eigenvector (Figure 9B–E). As a result, 13 MRGs were identified as hubs using the intersection of the top half of the genes with high topological properties (Figure 9F). The expression of five of these genes (Supplementary Figure S5), *ACSL1, NDUFA2, CYP1B1, SLC11A1*, and *COX6B1*, correlated with survival outcomes (Figure 9G–K). Ultimately, our study identified a total of five genes as potential biomarkers associated with MRG score, revealing their potential significance in clinical applications.

### Expression of *ACSL1, NDUFA2, CYP1B1, SLC11A1*, and *COX6B1* in GBM tissues

**Figure 10:**
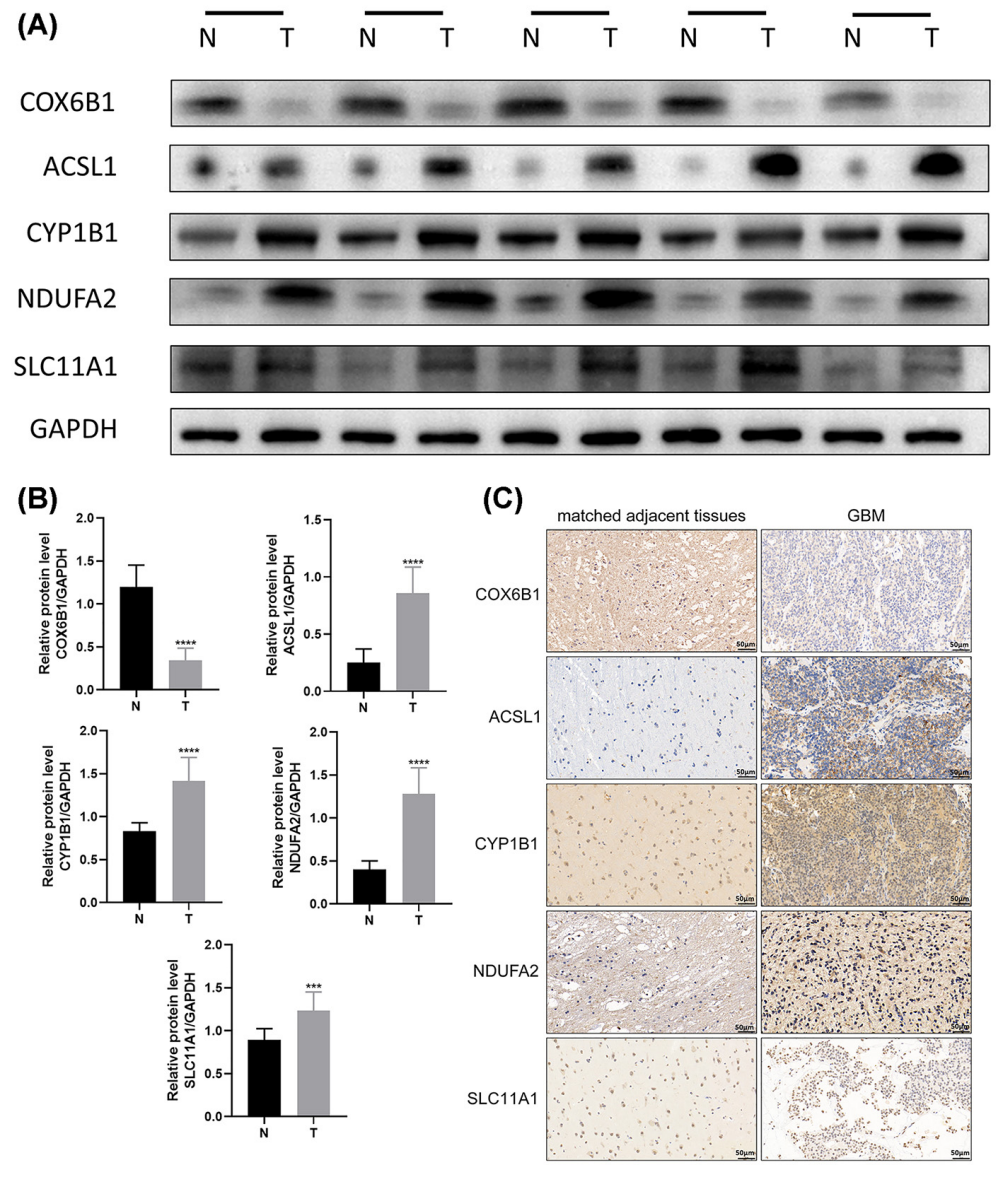
Expression of related genes in GBM tissues (**A**) The protein expression of *COX6B1, ACSL1, CYP1B1, NDUFA2*, and *SLC11A1* in GBM tissues. (**B**) Quantification of protein expression. (**C**) Immunohistochemistry of the expression of the five genes in GBM cases. Representative images of IHC staining show the expression of *COX6B1, ACSL1, CYP1B1, NDUFA2*, and *SLC11A1* in tumor tissues (right panel) and the matched adjacent tissues (left panel). ***P<0.001; ****P<0.0001; GBM, glioblastoma.

To confirm the roles of *ACSL1, NDUFA2, CYP1B1, SLC11A1*, and *COX6B1*, GBM tissues and corresponding adjacent tissue samples from 10 patients were used for testing. The expression levels of *ACSL1, NDUFA2, CYP1B1*, and *SLC11A1* were significantly higher in GBM tissues compared with those in matched adjacent tissues (Figure 10A,B). Conversely, the expression of *COX6B1* was higher in the matched adjacent tissues. We performed immunohistochemical staining for these five genes to further validate these findings. The staining patterns of the tumor tissues and matched adjacent tissues are shown in Figure 10C. Furthermore, the corresponding protein expression levels of these genes were consistent with this bioinformatic data, further confirming the role of these genes in tumor development.

### The influences of changes in *ACSL1, NDUFA2, CYP1B1, SLC11A1*, and *COX6B1* expression on GBM cell lines function

**Figure 11:**
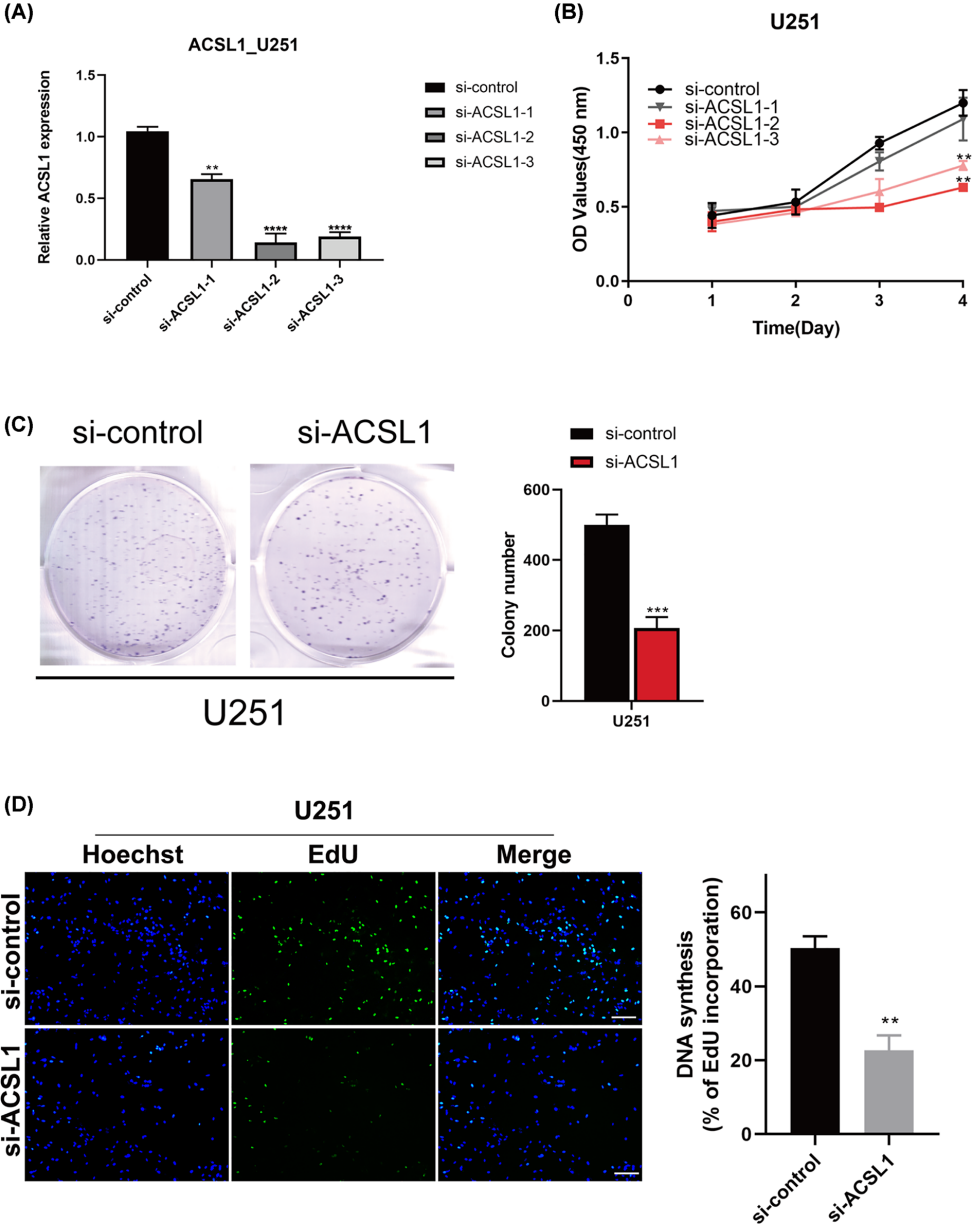
Down-regulation of *ACSL1* suppresses proliferation *in vitro* (**A**) Silencing efficiency of *ACSL1* in U251. (**B**) Cell viability assay of U251. (**C**) Colony formation of U251. (**D**) EdU assay of U251; **P<0.01, ***P<0.001, ****P<0.0001.

**Figure 12:**
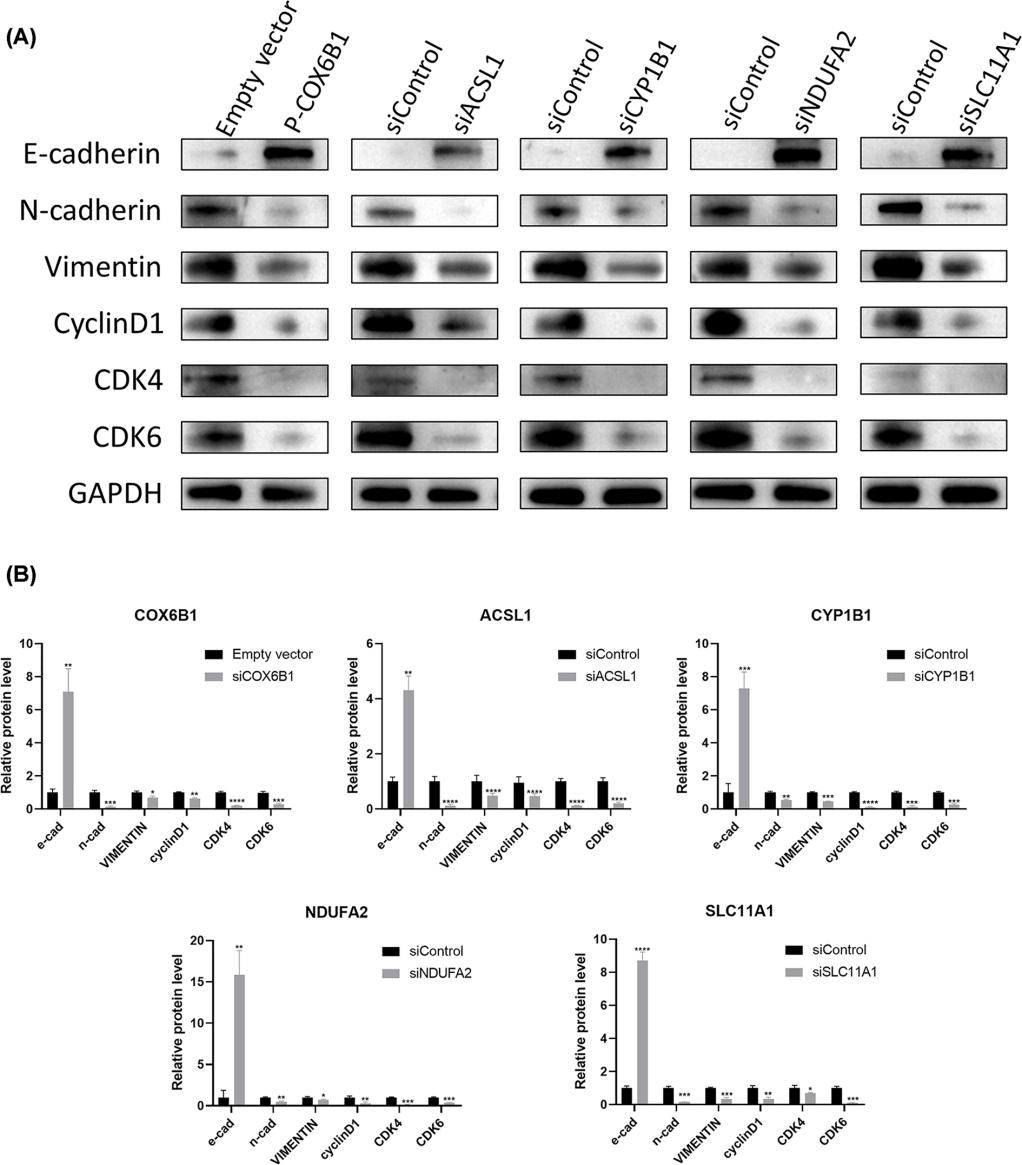
Effects on the endothelial-mesenchymal transition and cell cycle of GBM cells after regulating the expression of five genes (**A**) The protein expression of E-cadherin, N-cadherin, vimentin, cyclinD1, CDK4, and CDK6. (**B**) Quantification of (A); *P<0.05, **P<0.01, ***P<0.001, ****P<0.0001; GBM, glioblastoma.

*ACSL1, NDUFA2, CYP1B1, SLC11A1*, and *COX6B1* were expressed in GBM cells. U251 and U87 cell lines were chosen for functional verification experiments (Supplementary Figure S6). First, three types of siRNAs were designed to knock down the expression of *ACSL1, NDUFA2, CYP1B1*, and *SLC11A1*, and the plasmid was used to overexpress *COX6B1*. RT-qPCR was used to determine the transfection efficiency and western blot was used to detect protein level of *ACSL1, NDUFA2, CYP1B1, SLC11A1* and *COX6B1* (Supplementary Figure S7). Second, the effects of the five genes on cell proliferation were investigated using CCK-8 and EDU assays and colony formation experiments. As shown in Figure 11 and Supplementary Figures S8–S11, down-regulation of *ACSL1, NDUFA2, CYP1B1*, and *SLC11A1* and up-regulation of *COX6B1* markedly promoted the proliferation of GBM cells. Third, Western blotting experiments demonstrated that interventions in gene expression decreased the level of E-cadherin and increased that of N-cadherin and vimentin in the GBM cell lines (Figure 12). Moreover, the expression levels of cyclin D1, CDK4, and CDK6 were down-regulated. These results indicate that *ACSL1, NDUFA2, CYP1B1, SLC11A1*, and *COX6B1* are potentially related to endothelial-mesenchymal transition and cell cycle pathways in GBM cells.

## Discussion

The metabolism of cancer cells is a potent factor in the tumor immune microenvironment, and the alteration of the metabolism is considered to be involved in cancer cells’ somatic evolution, metastasis, therapeutic response, and others [9,10]. The metabolism within tumors is heterogeneous [35]. This heterogeneity has important associations with prognosis, tumor staging, and immunotherapy of patients with cancer. The heterogeneity in metabolic pathways and the relationship between the pathways and molecular subtypes as well as prognosis have been reported in various tumors [24,36,37], but have not been reported in detail in GBM. To identify the GBM subtypes associated with metabolic processes, GBM classification was established in this study based on 2752 MRGs screened from previous publications [16]. Through a cluster analysis of 153 GBM samples from the RNA-sequencing data of TCGA-GBM, three metabolism subtypes of GBM, including MC1, MC2, and MC3, were identified. In further analysis, we explored the prognosis value, metabolic signatures, immune infiltration, and immunotherapy sensitivity of the metabolism subtypes. Besides, the metabolism scoring model was established to measure the different metabolic characteristics of the patients, and five potential MRGs were identified based on metabolism-related co-expression module analysis. Laboratory-based validation tests further showed the expression of these MRGs in GBM tissues and how their expression influences cell function.

We identified three metabolism GBM subtypes, named MC1, MC2, and MC3. These subtypes categorize GBM patients into groups with varying metabolic characteristics. The identified metabolism subtypes could potentially serve as valuable prognostic indicators for GBM patients, and may have implications for the development of targeted therapies tailored to specific metabolic subtypes. Notably, the MC1 subtype has 37 specific metabolic characteristics, the most compared with the other two subtypes. Additionally, it can thus be considered a metabolically active subtype. The hypermetabolic activity of MC1 cells can induce changes in the tumor microenvironment and result in hypoxia, nutrient depletion, acidity, and the generation of metabolites that can be toxic at increased concentrations [32]. These changes may, in turn, suppress the anti-tumor immune response and worsen the prognosis. For example, glycolysis, a specific metabolic characteristic of MC1, promotes GBM cell proliferation and tumorigenesis [38], and this is of great importance for the treatment strategies of patients with the MC1 subtype. Increased glycolysis is a critical hallmark of cancer, and it can promote cancer cell proliferation, aggressiveness, and drug resistance [7], and this might be the reason why MC1 has the worst prognosis. In addition, MC1 showed the lowest TIDE score, suggesting that patients with the MC1 subtype may benefit the most from immunotherapy. Therefore, the hypermetabolic activity of the MC1 subtype could provide the basis for developing new therapeutic strategies based on the modulation of metabolism, and this would be very significant. Most metabolic characteristics of the MC2 subtype were associated with lipid, amino acid, and nucleotide metabolism. The results showed an obvious survival advantage for patients with the least-specific metabolic characteristics of the MC3 subtype. One of these, oxidative phosphorylation, was previously implied to play a significant role in the survival of patients with GBM [39]. These findings contribute to a comprehensive understanding of the distinctive metabolic profiles within GBM subtypes and their implications for patient prognosis and therapeutic interventions.

To comprehensively understand the distinct metabolic subtypes of GBM, we investigated multiple aspects in addition to metabolic characteristics. In terms of clinical characteristics, the MC1 subtype had a higher proportion of patients younger than 60 than the MC2 subtype. This finding aligns with the conclusions obtained in a previous study, in which the 10-year survival rate was inversely related to age at diagnosis [40]. IDH mutations are considered the main determinants of the genomic landscape and, thus, biomarkers for subtype classification in diffuse gliomas [41]. A previous study [42] showed that IDH mutations predicted favorable disease outcomes with prolonged median survival in GBM. Similarly, we showed that the MC3 subtype contained the highest proportion of patients with IDH mutations and identified an association between MC3 and favorable survival outcomes.

Analyzing mutation profiles and heterogeneity of tumors can help identify therapeutic targets and predict drug sensitivity, offering support for precision medicine practices [43,44]. Among these mutational profiles, Homologous recombination is a crucial DNA lesion repair mechanism [45]. Our results showed that homologous recombination defects were higher in the MC1 subtype than in the other MC2 subtypes. A previous study [46] highlighted the contribution of homologous recombination defects to radioresistance in glioma stem cells, which may play a role in GBM recurrence. Tumor purity and proliferation in the MC3 subtype were significantly lower than in the MC1 or MC2 subtypes. Low tumor purity and related cellular heterogeneity are associated with an aggressive phenotype and poor prognosis in gliomas [47], and this is not in line with our findings, which may be due to discrepancies in the surgical sampling of the tissues [48]. As previously mentioned, the stemness index, which includes mRNAsi and EREG-mRNAsi, was used to investigate molecular heterogeneity within tumors [27]. mRNAsi, reflecting gene expression, was significantly associated with tumor histologic grade and overall survival in patients with glioma [49]. Survival duration is longer in patients with lower-grade gliomas and low mRNAsi. This conclusion is consistent with our findings, which showed that the mRNAsi of the MC1 subtype was significantly lower than that of other subtypes. EREG-mRNAsi is a stemness index generated using a set of stemness-related epigenetically regulated genes. In the previous study, the higher EREG-mRNAsi group had a higher mortality rate than the lower group [50]. However, our findings were in the opposite direction. The MC1 subtype, which had a smaller EREG-mRNAsi, showed a worse prognosis. These results presented the complex interplay of metabolism subtypes, both with mutation profiles and heterogeneity, in influencing patient outcomes, warranting further investigation into the underlying mechanisms.

The tumor microenvironment, consisting of tumor cells and adjacent non-tumor cells [51], plays a crucial role in tumor biology. Scores of immune and stromal cells, the major components of the tumor microenvironment [52], were previously shown to be associated with GBM subtypes. Hence, we evaluated immune cell infiltration using five different algorithms between different metabolism subtypes of GBM in the present study. The EPIC method showed a significantly higher proportion of CD4^+^ T cells in the MC3 subset than in other subtypes. CD4^+^ T cells were previously demonstrated to promote anti-tumor immunity and enhance immunotherapy through multiple pathways [53–55]. These results suggest that CD4^+^ T cells are associated with an improved prognosis, which aligns with our findings. The ESTIMATE evaluation showed that the MC1 subtype had the highest ImmuneScore, StromalScore, and ESTIMATEScore. The median survival of the low-score group was reported to be longer than that of the high-score group [56,57]. Here, the MC1 subtype yielded the highest score and was associated with poor survival. In contrast, the MC2 subtype yielded the highest TIDE score, suggesting that patients with this subtype are less likely to benefit from immunotherapy. These results indicate a higher-than-expected complexity of the anti-tumor function of immune cell variation in diffuse gliomas. This comprehensive analysis of GBM metabolism subtypes reveals the complexity of GBM metabolism and is of significant importance for the development of targeted treatments and personalized therapeutic strategies. The distinct metabolic profiles, genetic traits, and immune characteristics discerned within each subtype hold potential as biomarkers for patient stratification, guiding the choice of suitable therapeutic interventions. Furthermore, a deeper understanding of the characteristics of these different metabolism subtypes identified in the present study could provide valuable insights into the potential molecular mechanisms driving GBM progression, and potentially inform the development of new treatment methods aimed at each subtype. Further research is required to validate the functional relevance of these identified distinct features and their potential contribution to the pathogenesis of GBM. The ultimate goal is to translate these findings into clinical applications that improve patient prognosis.

In order to better quantify the metabolism-related characteristics, we developed an MRG score. The development of MRG score can offer precise data guidance for clinical decision-making, thereby translating research findings into clinically meaningful guidance. Furthermore, we analyzed the correlation between the MRG score and immune infiltration and metabolic processes. Accordingly, the enrichment scores of most immune cells and the MRG score of the MC1 subtype were higher than those of other subtypes. Further analysis revealed that the MRG score was significantly and positively correlated with immature dendritic cells, NK T cells, central memory CD4 T cells, central memory CD8 T cells, and plasmacytoid dendritic cells and negatively correlated with CD56dim NK cells, monocytes, and type 17 T-helper cells. We identified a positive correlation between the MRG score and metabolic pathways involved in galactose, retinoic acid, and retinol metabolism. In contrast, a negative correlation was identified between the MRG score and the metabolic pathways of pyrimidine biosynthesis, glyoxylate, and dicarboxylate metabolism. The driving factors behind these correlations are currently unclear and require further research into their underlying mechanisms.

Based on co-expression network analysis, we identified five MRGs (*COX6B1, ACSL1, NDUFA2, CYP1B1*, and *SLC11A1*) whose expression correlated with survival outcomes. These five genes are potential biomarkers associated with the MRG score. Laboratory tests were performed to validate this correlation. The results revealed the significant roles of *COX6B1, ACSL1, NDUFA2, CYP1B1*, and *SLC11A1* in GBM, with the protein expression levels consistent with bioinformatic data and alterations resulting in changes in cell proliferation. The identified genes could potentially be linked to the endothelial-mesenchymal transition pathway and the cell cycle in GBM cells, implying that manipulation of these genes could lead to innovative therapeutic strategies. Our findings suggest a new avenue for disrupting tumor progression and highlight the scope for targeted gene therapy in GBM management. Most of the previous studies on these genes were not focused on GBM. *COX6B1*, a mitochondrial gene, was previously reported to be associated with encephalomyopathy, hydrocephalus, and cardiomyopathy [58,59]. However, *COX6B1* has rarely been highlighted in tumors. Since *COX6B1* is the only protective gene investigated here, further research into the associations of this gene with GBM may be highly beneficial. *ACSL1*, associated with lipid metabolism [60], is an oncogene whose significance in many cancers is well established [61]. *NDUFA2* is a mitochondrial gene [62]. Wang et al. [62] reported an increased expression of *NDUFA2* in the cytoplasm of paraganglioma cells. *CYP1B1* is involved in the metabolism of xenobiotics and endogenous substances and exhibits higher expression in tumor cells than in the surrounding normal tissues [63]. A previous study indicated that *SLC11A1* can modulate macrophage activation [64]. The results advanced our understanding of the molecular mechanisms governing GBM proliferation and development. These genes may be used as biomarkers to predict treatment outcomes and patient prognosis [65], and the functional role of these genes deserves further investigation.

The lack of clinical data and supporting experimental evidence were major limitations of this study. Although we validated the GBM metabolism subtype classification in independent cohorts, additional clinical data may still be required to establish a more reliable classification procedure. Moreover, the biological functions of the five MRGs need to be explored in future experimental studies.

## Conclusions

In conclusion, we classified patients with GBM into three metabolism subtypes: MC1, MC2, and MC3. The MC1 subtype has the worst prognosis and the most metabolic characteristics among them. More studies regarding therapeutic strategies targeting cancer metabolism, especially for patients of the MC1 subtype, are warranted. These findings have implications for future research and treatment, particularly for the five MRGs and their role in survival outcomes. Understanding the metabolic and immunological characteristics of the GBM subtypes identified here will facilitate the development of new clinical management and treatment strategies.

## Supplementary Material

Supplementary Figures S1-S11 and Tables S1-S3

## Data Availability

The data generated in this study are available may be requested from the corresponding authors.

## Abbreviations

CDF: cumulative distribution function
CGGA: Chinese Glioma Genome Atlas
GBM: glioblastoma
IDH: isocitrate dehydrogenase
MRG: metabolism-related gene
TCGA: The Cancer Genome Atlas
