# Supplementary material for: Integrative analysis of metabolism subtypes and identification of prognostic metabolism-related genes for glioblastoma
Source: Biosci Rep. 2024 Mar 22;44(3):BSR20231400. doi: 10.1042/BSR20231400 (PMC10965397; doi:10.1042/BSR20231400)
Supplement: Supplementary Figures S1-S11 and Tables S1-S3 [file BSR-2023-1400_supp.pdf]

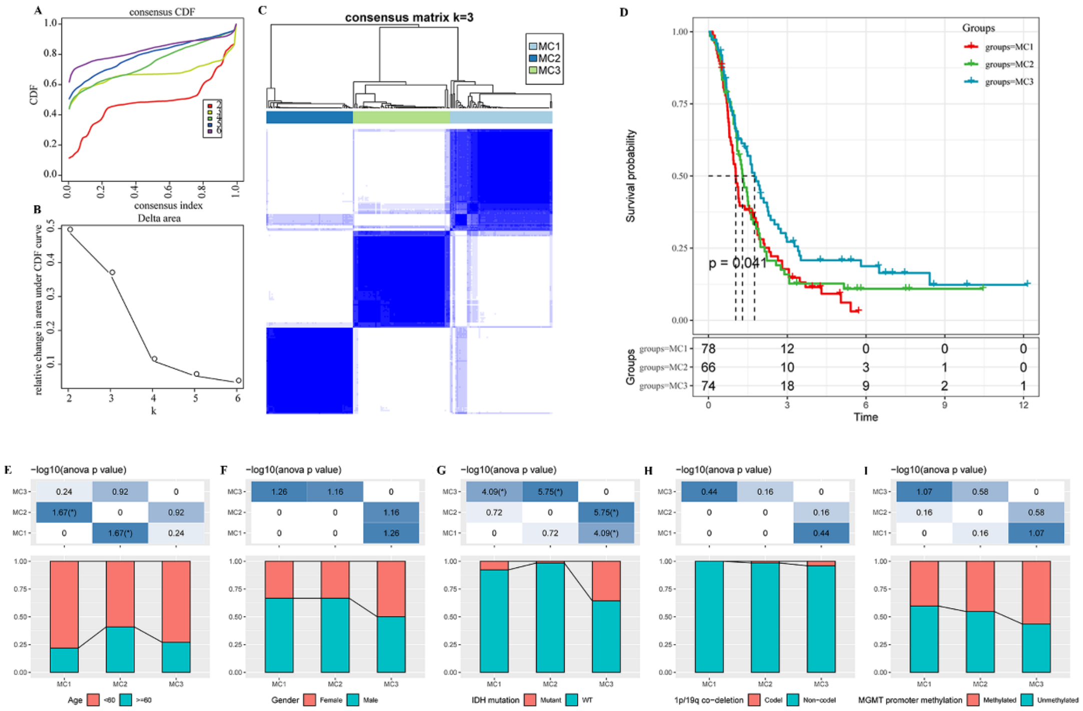

Figure S1. Metabolism subtypes of GBM. (A) CDF curve based on CGGA-GBM cohort data. (B) CDF delta area curve based on the CGGA-GBM cohort data. (C) Clustering heatmap based on data from the CGGA-GBM samples with consensus k = 3. (D) Kaplan–Meier curve for overall survival among subtypes in the CGGA cohort. (E) Age distribution of metabolism subtypes in the CGGA cohort. (F) Gender distribution of metabolism subtypes in the CGGA cohort. (G) IDH mutation status distribution of metabolism subtypes in the CGGA cohort. (H) 1p/19q codeletion status distribution of metabolism subtypes in the CGGA cohort. (I) MGMT promoter methylation of metabolism subtypes in the CGGA cohort. *p <0.05. CDF, cumulative distribution function; GBM, glioblastoma; CGGA, Chinese Glioma Genome Atla; IDH, isocitrate dehydrogenase; MGMT, O6-methylguanine-DNA methyltransferase

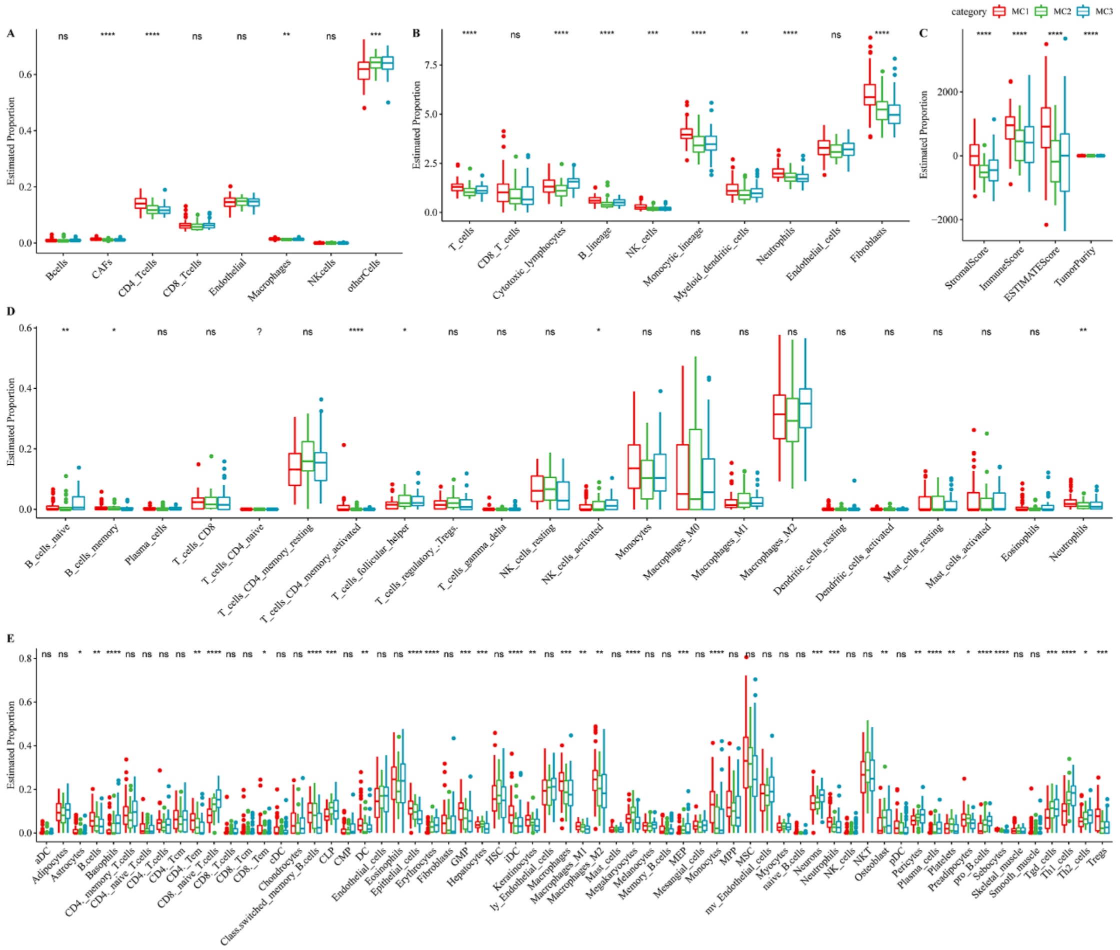

Figure S2. Immune cell proportions across metabolism subtypes. (A) Immune cell proportions calculated using EPIC method. (B) Immune cell proportions calculated using MCPcounter. (C) Immune cell proportions calculated using ESTIMATE. (D) Immune cell proportions calculated using CIBERSORT. (E) Immune cell proportions calculated using xCell method.

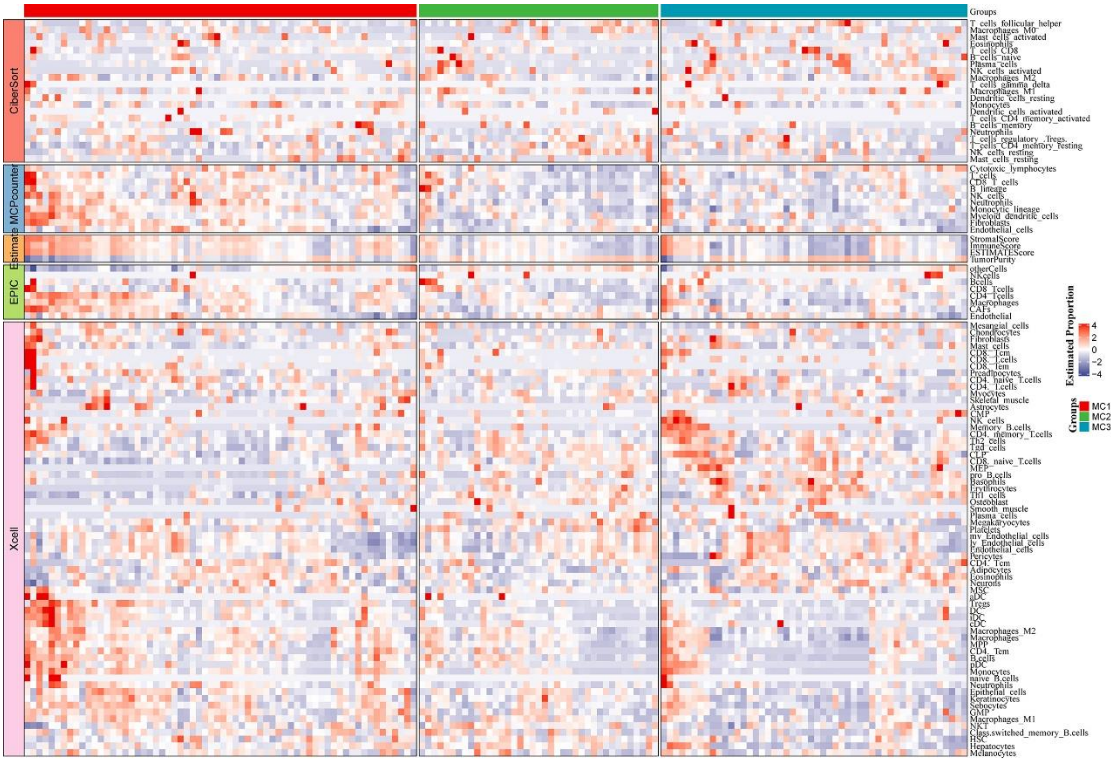

Figure S3. Heatmap of immune-associated genes constructed using immune infiltration software (EPIC, MCPcounter, ESTIMATE, CIBERSORT, xCell) in the TCGA-GBM cohort

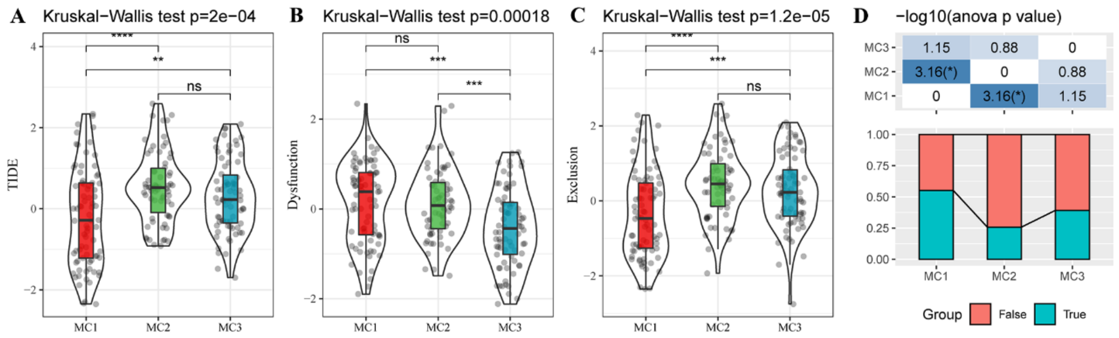

Figure S4. Immunotherapy response across metabolism subtypes. (A) TIDE scores of metabolism subtypes in the CGGA cohort. (B) T-cell dysfunction scores of metabolism subtypes in the CGGA cohort. (C) T-cell exclusion scores of metabolism subtypes in the CGGA cohort. (D) Predicted immunotherapy response status of metabolism subtypes in the CGGA cohort. CGGA, Chinese Glioma Genome Atlas

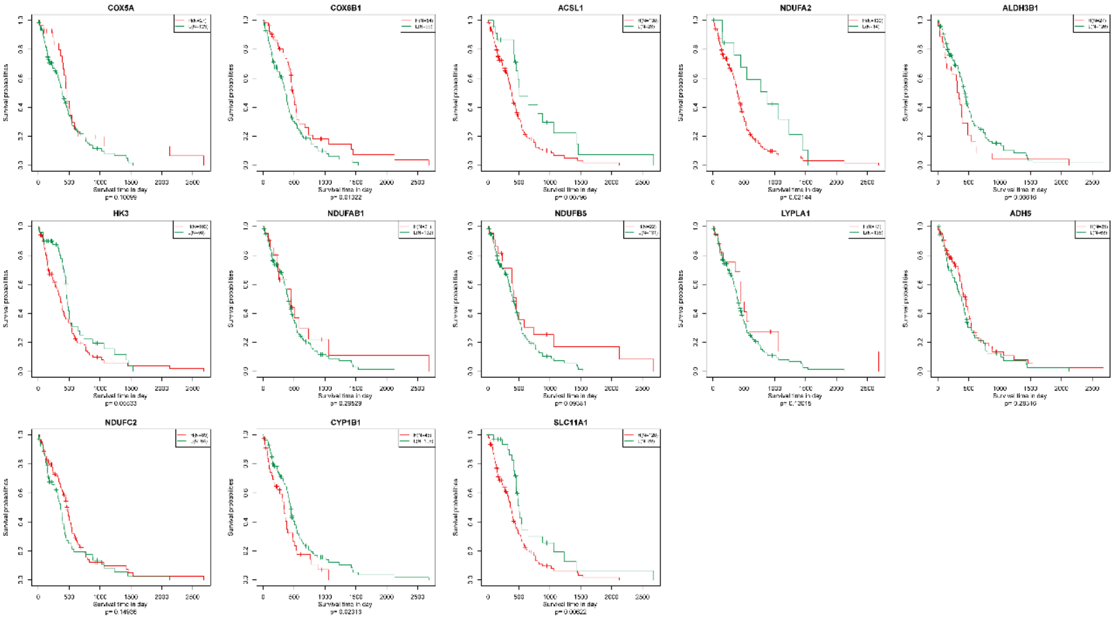

Figure S5. The Kaplan–Meier survival curves of 13 hub MRGs

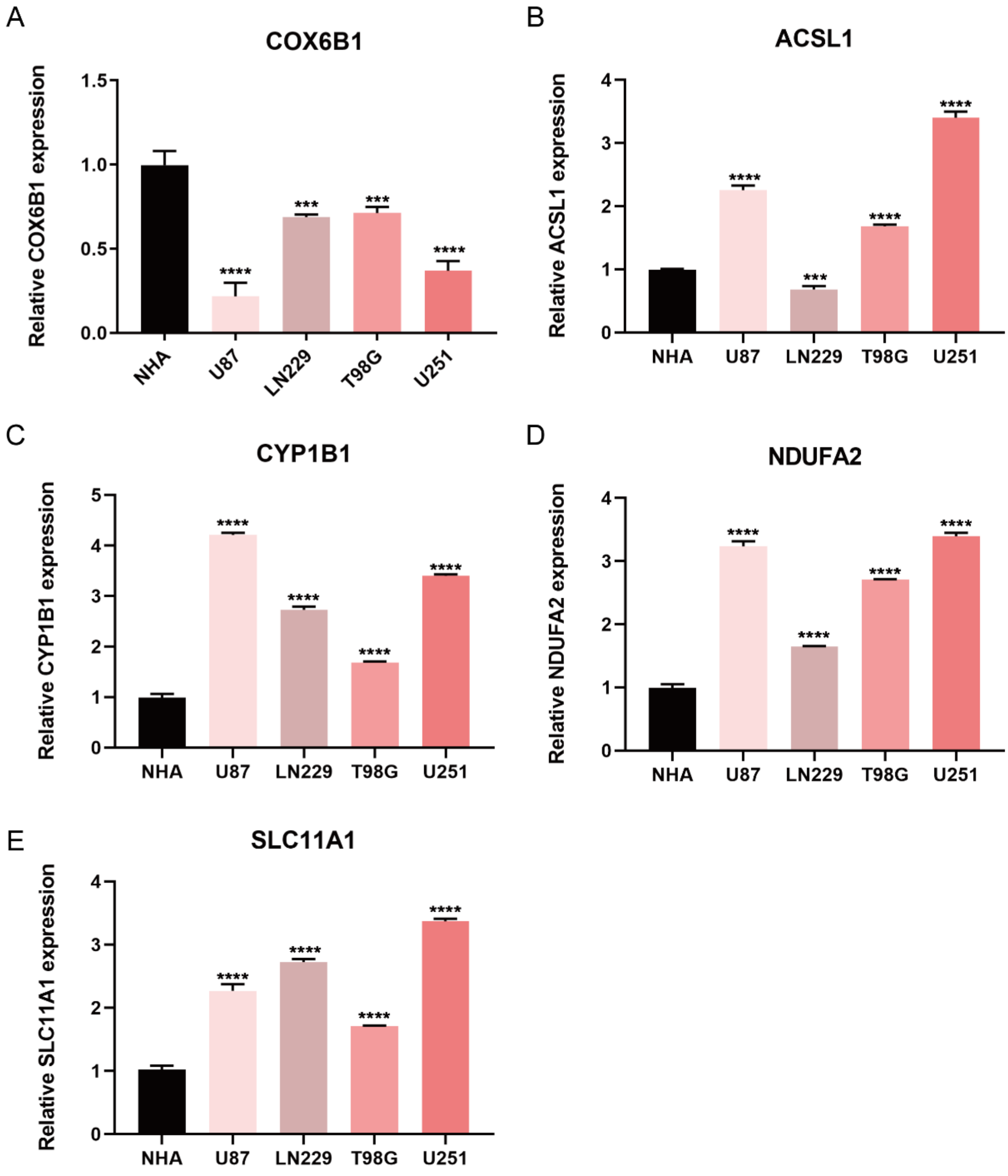

Figure S6. RT-qPCR was used to detect the expression of the 5 MRGs in GBM cell lines. (A) the expression of COX6B1. (B) the expression of ACSL1. (C) the expression of CYP1B1. (D) the expression of NDUFA2. (E) the expression of SLC11A1.

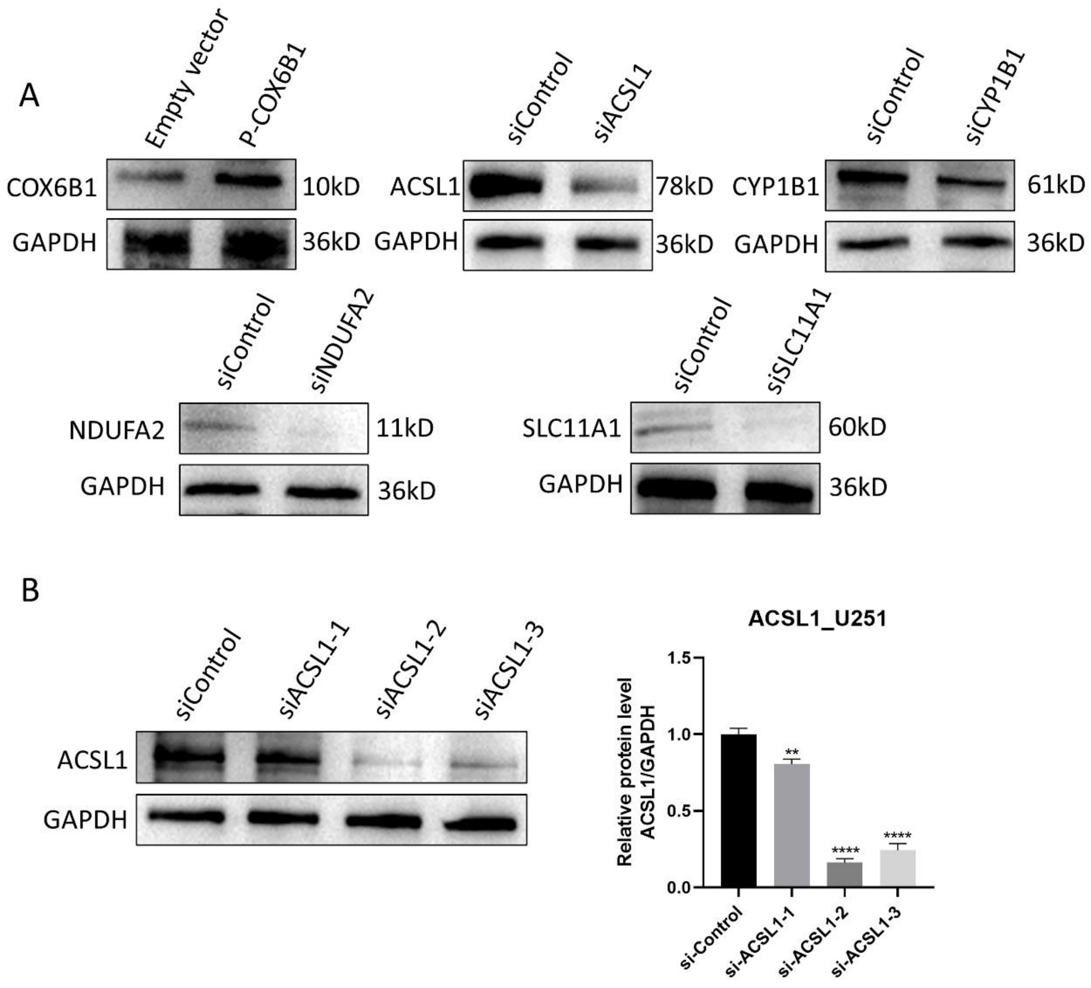

Figure S7. The knockdown and overexpression efficiency of proteins. (A) The knockdown and overexpression efficiency of each protein in Figure 12. (B) The knockdown efficiency of ACSL1 in U251. **p <0.01; ***p <0.001; ****p <0.0001.

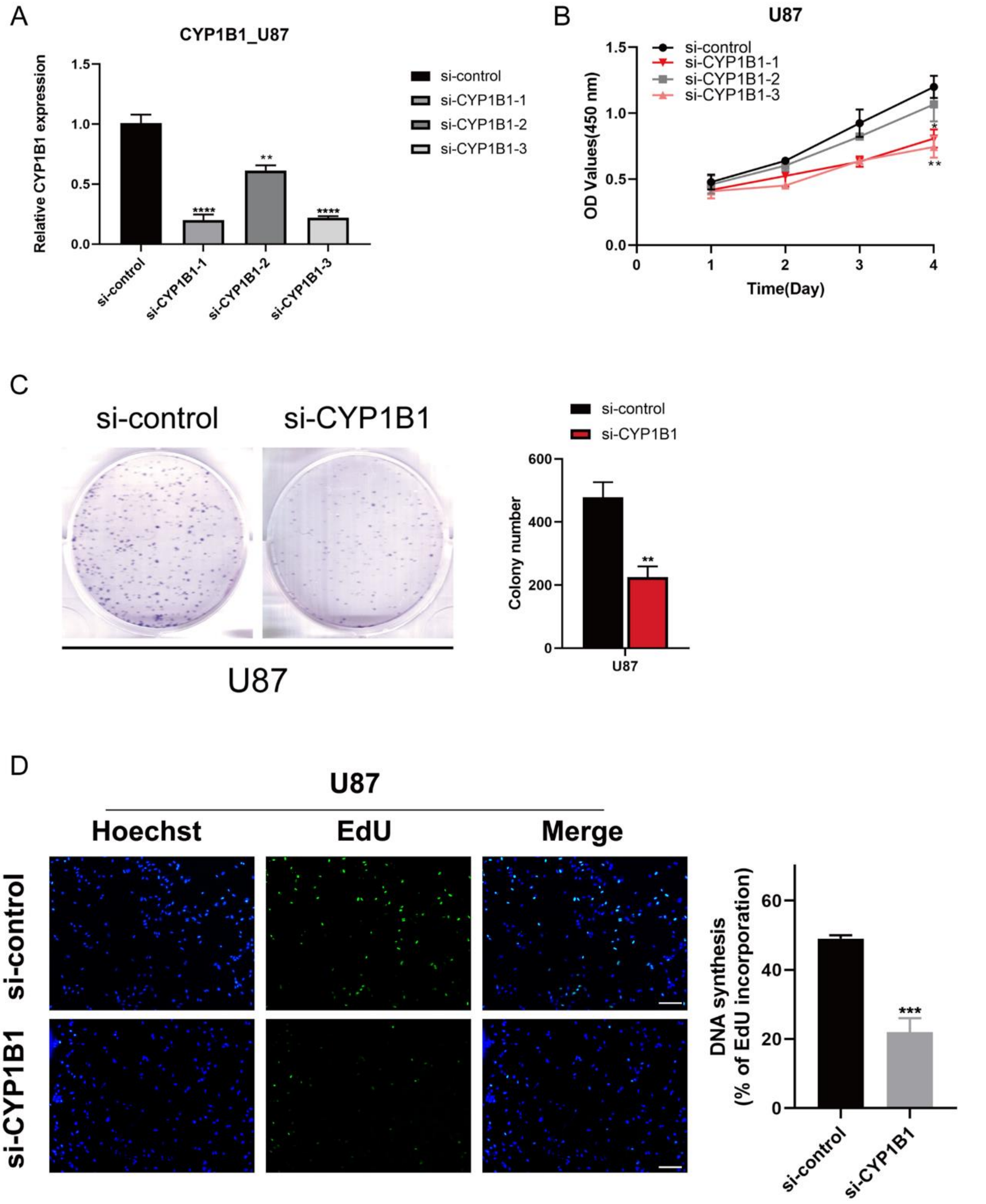

Figure S8. Downregulating CYP1B1 suppresses proliferation in vitro. (A) Silencing efficiency of CYP1B1 in U87. (B) Cell viability assay of U87. (C) Colony formation of U87. (D) EdU assay of U87. **p <0.01; ***p <0.001; ****p <0.0001.

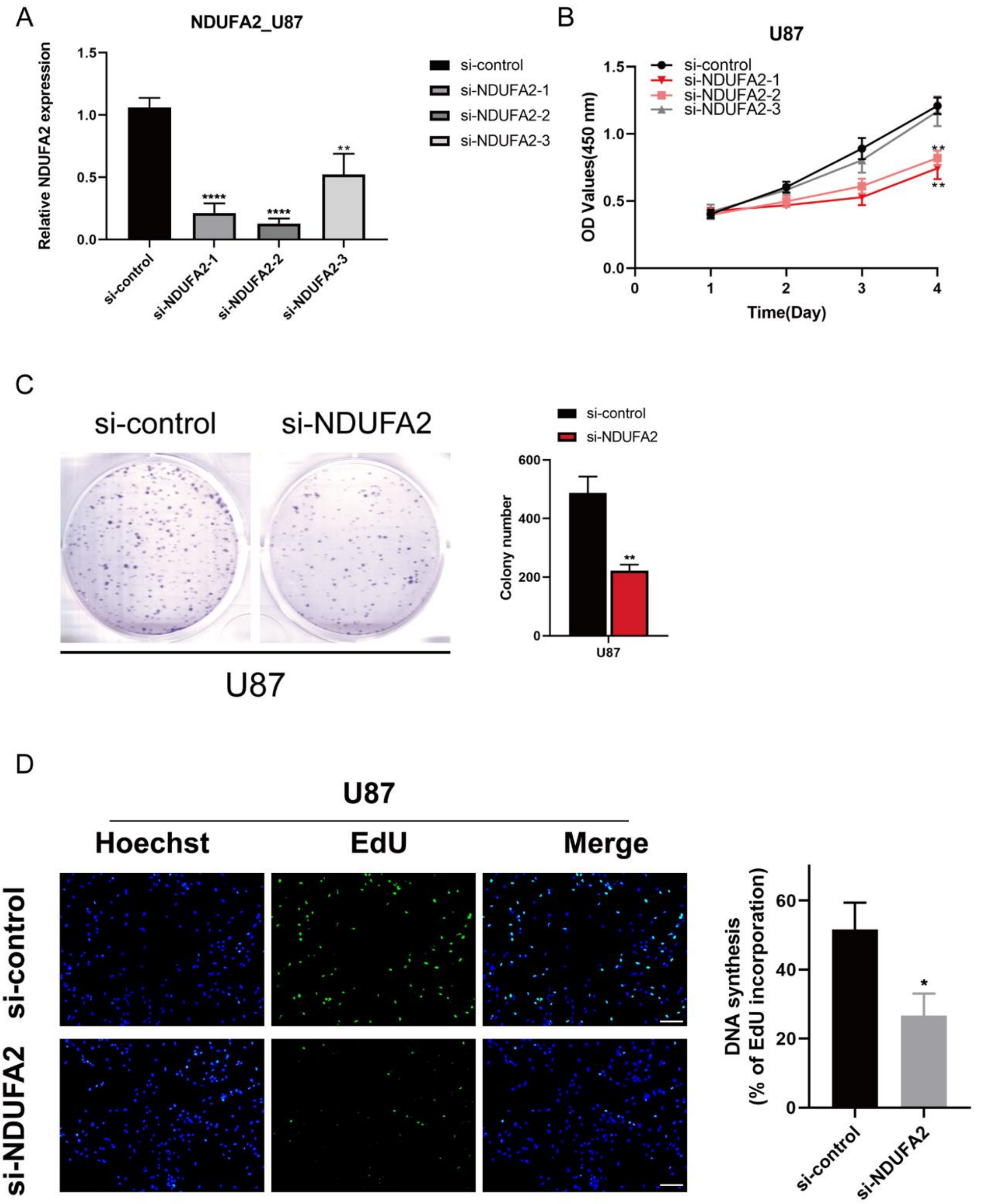

Figure S9. Downregulating NDUFA2 suppresses proliferation in vitro. (A) Silencing efficiency of NDUFA2 in U87. (B) Cell viability assay of U87. (C) Colony formation of U87. (D) EdU assay of U87. *p <0.05; **p <0.01; ***p <0.001; ****p <0.0001.

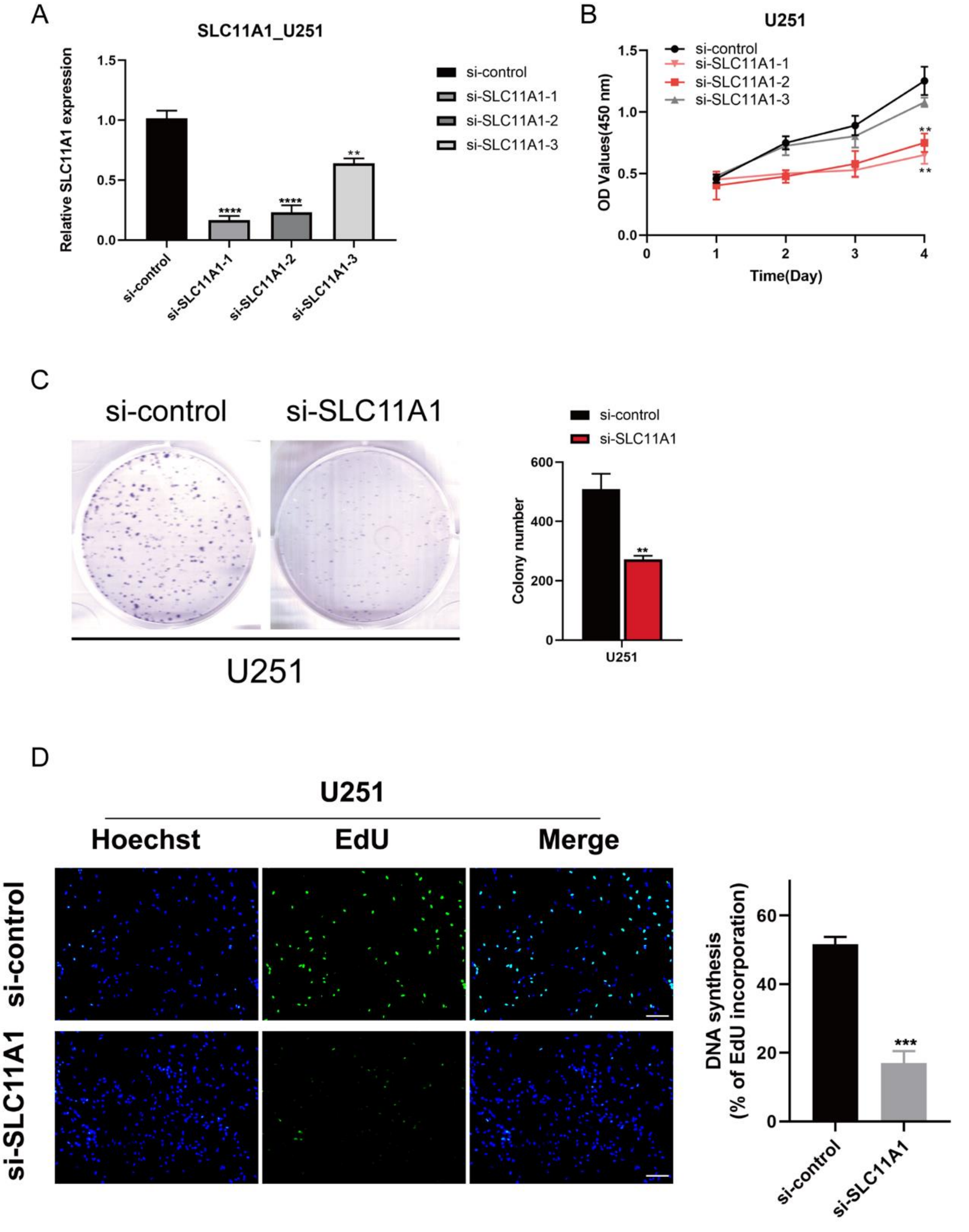

Figure S10. Downregulating SLC11A1 suppresses proliferation in vitro. (A) Silencing efficiency of SLC11A1 in U251. (B) Cell viability assay of U251. (C) Colony formation of U251. (D) EdU assay of U251. **p <0.01; ***p <0.001; ****p <0.0001.

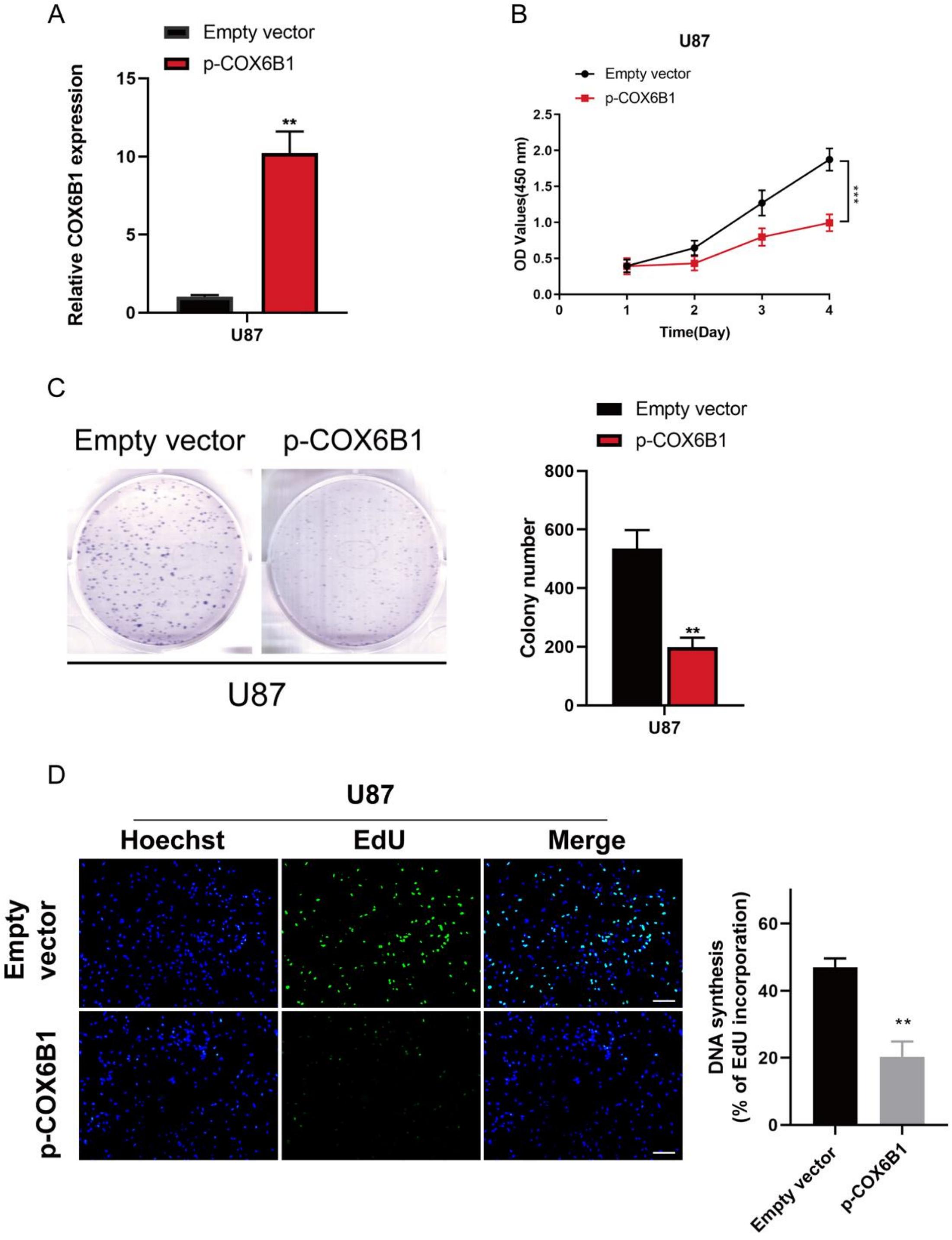

Figure S11. Upregulating COX6B1 suppresses proliferation in vitro. (A) Silencing efficiency of COX6B1 in U87. (B) Cell viability assay of U87. (C) Colony formation of U87. (D) EdU assay of U87. *p <0.05; **p <0.01; ***p <0.001.

Table S1. The qRCR related primers

Gene	Forward primer	Reverse primer
ACSL1	GTGGAACTACAGGCAACCCC	ATCATCTGGGCAAGGATTGAC
NDUFA2	AAACGCTACGTGGAGCTGAA	TGCACCCTGGAGGCGTA
CYP1B1	TCCTCCTCTTCACCAGGTATCC	TGGTCACCCATACAAGGCAG
SLC11A1	TTACTCACTCGGACCAGCAC	GGGGGCTCTTGTCACTAATCA
COX6B1	CGGGGTGCCTTTAGGATTCA	TTCTGACAGCGGTGGAAGTC

Table S2. Target sequences of small interfering RNA

Gene	Target sequence
siACSL1-1	GGCCTTCATGTTGTTAATTTTGA
siACSL1-2	AAGATCATGTTGGGATTAACTTG
siACSL1-3	AAGCATTTCTTTAAAGCTATTAA
siNDUFA2-1	CTGGACTTAGTATAATGTGAAAA
siNDUFA2-2	CTCCTATTCCTCATAAAGCTTGT
siNDUFA2-3	AGGCAAAGTAGCTTAATATAAAA
siCYP1B1-1	CACCGTTTTCCGCGAATTCGAGC
siCYP1B1-2	AACCATTAAACCCAAGTCATTTA
siCYP1B1-3	AACTTAGACTCTAGTATTTATGG
siSlc11a1-1	TCCAGAACTATGCTAAGATCTTC
siSlc11a1-2	CGCCATCAACCTGTACTTTGTGA
siSlc11a1-3	TTCAGAAGATGGGGATTCAATTC

Table S3. The interaction confidence score of the protein–protein interaction network

Gene1	Gene2	score
ACSL1	ADH5	163
ACSL1	ATP8B4	185
ACSL1	COX6B1	187
ACSL1	CYP1B1	215
ACSL1	DGKI	213
ACSL1	FMO1	211
ACSL1	KYNU	392
ACSL1	LYPLA1	162
ACSL1	MGAT1	162
ACSL1	NDUFA2	165
ACSL1	NDUFA4	233
ACSL1	NDUFAB1	266
ACSL1	NDUFB5	177
ACSL1	PLB1	171
ACSL1	PLCG2	156
ACSL1	PPA2	222
ACSL1	SLC11A1	168
ACSL1	TBXAS1	218
ADH5	ALDH3B1	799
ADH5	CYP1B1	195
ADH5	HK3	166
ADH5	IAH1	362
ADH5	MICAL3	227
ADH5	SDHD	229
ADH5	SLCO2B1	176
ADH5	SUCLG1	159

ADH5	TRPM2	173
ALDH3B1	ATP8B4	903
ALDH3B1	CYBB	907
ALDH3B1	CYP1B1	479
ALDH3B1	FMO1	186
ALDH3B1	HGSNAT	902
ALDH3B1	HK3	209
ALDH3B1	KYNU	223
ALDH3B1	MFSD1	284
ALDH3B1	NDUFA2	349
ALDH3B1	NDUFAB1	382
ALDH3B1	SLC2A5	908
ALDH3B1	SUCLG1	192
ALDH3B1	TRPM2	900
ALOX5	ALOX5AP	997
ALOX5	AOAH	224
ALOX5	CYBB	460
ALOX5	CYP1B1	169
ALOX5	HGSNAT	250
ALOX5	HK3	334
ALOX5	PIK3R5	300
ALOX5	PLB1	684
ALOX5	SLC11A1	184
ALOX5	SLC7A7	241
ALOX5	SLCO2B1	346
ALOX5	TBXAS1	665
ALOX5	TRPM2	151
ALOX5	TYMP	155
ALOX5AP	AOAH	371

ALOX5AP	CYBB	371
ALOX5AP	HK3	353
ALOX5AP	PIK3R5	213
ALOX5AP	PLCG2	152
ALOX5AP	SLC11A1	305
ALOX5AP	SLC7A7	328
ALOX5AP	SLCO2B1	236
ALOX5AP	TBXAS1	525
AOAH	ATP8B4	165
AOAH	CYBB	280
AOAH	HK3	526
AOAH	MGAT4A	171
AOAH	PIK3R5	213
AOAH	SLC11A1	155
AOAH	SLC7A7	315
AOAH	ST3GAL1	222
AOAH	STARD13	169
AOAH	TBXAS1	225
AOAH	TYMP	151
ASNA1	NDUFAB1	222
ASNA1	NDUFB9	181
ASNA1	UQCRQ	177
ATP11C	ATP8B4	152
ATP11C	SLC11A1	241
ATP8B4	CYBB	913
ATP8B4	HGSNAT	928
ATP8B4	HK3	282
ATP8B4	SCN3A	170
ATP8B4	SLC11A1	931

ATP8B4	SLC2A5	913
ATP8B4	TRPM2	924
CACNA2D4	CYBB	151
CACNA2D4	HK3	159
CACNA2D4	PIK3R5	152
CACNA2D4	SCN3A	311
CHSY1	DSE	554
CHSY1	GBGT1	304
CHSY1	HGSNAT	287
COQ5	COX5A	150
COQ5	COX6A1	213
COQ5	COX6B1	251
COQ5	KYNU	208
COQ5	NDUFA12	182
COQ5	NDUFAB1	240
COQ5	NDUFB6	239
COQ5	NDUFS4	182
COQ5	SUCLG1	211
COQ5	UQCRH	218
COQ5	UQCRQ	369
COQ5	VDAC3	167
COX5A	COX5B	999
COX5A	COX6A1	998
COX5A	COX6B1	999
COX5A	COX7B	989
COX5A	CYP1B1	157
COX5A	NDUFA12	964
COX5A	NDUFA2	821
COX5A	NDUFA4	967

COX5A	NDUFAB1	988
COX5A	NDUFAF2	197
COX5A	NDUFB5	981
COX5A	NDUFB6	973
COX5A	NDUFB9	968
COX5A	NDUFC2	929
COX5A	NDUFS3	984
COX5A	NDUFS4	976
COX5A	PPA2	316
COX5A	SDHD	819
COX5A	SLC11A1	286
COX5A	SUCLG1	726
COX5A	UQCRH	982
COX5A	UQCRQ	997
COX5A	VDAC3	588
COX5B	COX6A1	999
COX5B	COX6B1	999
COX5B	COX7B	993
COX5B	NDUFA12	940
COX5B	NDUFA2	745
COX5B	NDUFA4	977
COX5B	NDUFAB1	907
COX5B	NDUFB5	912
COX5B	NDUFB6	910
COX5B	NDUFB9	906
COX5B	NDUFC2	833
COX5B	NDUFS3	948
COX5B	NDUFS4	892
COX5B	PPA2	271

COX5B	SDHD	772
COX5B	SUCLG1	586
COX5B	UQCRH	941
COX5B	UQCRQ	998
COX5B	VDAC3	362
COX6A1	COX6B1	998
COX6A1	COX7B	978
COX6A1	NDUFA12	463
COX6A1	NDUFA2	485
COX6A1	NDUFA4	970
COX6A1	NDUFAB1	462
COX6A1	NDUFAF2	215
COX6A1	NDUFB5	597
COX6A1	NDUFB6	638
COX6A1	NDUFB9	649
COX6A1	NDUFC2	543
COX6A1	NDUFS3	616
COX6A1	NDUFS4	411
COX6A1	SDHD	480
COX6A1	SUCLG1	196
COX6A1	UQCRH	928
COX6A1	UQCRQ	992
COX6B1	COX7B	991
COX6B1	KYNU	432
COX6B1	NDUFA12	658
COX6B1	NDUFA2	650
COX6B1	NDUFA4	974
COX6B1	NDUFAB1	526
COX6B1	NDUFAF2	241

COX6B1	NDUFB5	759
COX6B1	NDUFB6	493
COX6B1	NDUFB9	712
COX6B1	NDUFC2	663
COX6B1	NDUFS3	688
COX6B1	NDUFS4	880
COX6B1	PPA2	189
COX6B1	SDHD	526
COX6B1	SUCLG1	269
COX6B1	TYMP	171
COX6B1	UQCRH	845
COX6B1	UQCRQ	989
COX6B1	VDAC3	364
COX7B	NDUFA12	497
COX7B	NDUFA2	769
COX7B	NDUFA4	985
COX7B	NDUFAB1	308
COX7B	NDUFAF2	358
COX7B	NDUFB5	619
COX7B	NDUFB6	764
COX7B	NDUFB9	610
COX7B	NDUFC2	539
COX7B	NDUFS3	394
COX7B	NDUFS4	542
COX7B	PPA2	282
COX7B	SDHD	500
COX7B	UQCRH	821
COX7B	UQCRQ	951
CYBB	GRIA2	254

CYBB	HGSNAT	903
CYBB	HK3	459
CYBB	PIK3R5	348
CYBB	PLCG2	157
CYBB	SLC11A1	950
CYBB	SLC2A5	907
CYBB	TBXAS1	195
CYBB	TRPM2	934
CYBB	TYMP	156
CYBB	VDAC3	150
CYP1B1	FMO1	421
CYP1B1	HK3	160
CYP1B1	KYNU	201
CYP1B1	SLCO2B1	153
CYP1B1	SLCO5A1	187
CYP1B1	SUCLG1	210
CYP1B1	TBXAS1	291
DGKI	GRIA2	201
DGKI	PLCG2	708
DGKI	TBXAS1	185
DPYSL4	DTYMK	162
DPYSL4	KYNU	221
DPYSL4	TYMP	150
DSE	HGSNAT	235
DSE	PIK3R5	451
DTYMK	IAH1	180
DTYMK	NDUFAB1	416
DTYMK	TYMP	519
FMO1	NDUFAB1	190

FMO1	SLC7A7	163
FMO1	SLCO2B1	157
FMO1	TBXAS1	261
GBGT1	MGAT1	284
GBGT1	MGAT4A	302
GBGT1	PPA2	151
GBGT1	ST3GAL1	568
GRIA2	GRIK2	636
GRIA2	LYPLA1	170
GRIA2	NPC2	179
GRIA2	SCN3A	409
GRIA2	TBXAS1	172
GRIK2	SCN3A	499
GRIK2	SUCLG1	230
HGSNAT	NDUFC2	262
HGSNAT	NPC2	299
HGSNAT	SLC11A1	900
HGSNAT	SLC2A5	900
HGSNAT	TRPM2	900
HK3	NDUFAB1	270
HK3	NPC2	189
HK3	PIK3R5	356
HK3	PLB1	155
HK3	SDHD	164
HK3	SLC11A1	573
HK3	SLC2A5	206
HK3	SLC7A7	213
HK3	SUCLG1	208
HK3	TBXAS1	285

HK3	TRPM2	216
HK3	TYMP	266
HK3	VDAC3	386
IAH1	NDUFAB1	231
KYNU	NDUFAB1	204
KYNU	SLC7A7	188
KYNU	TBXAS1	192
LYPLA1	NDUFA12	229
LYPLA1	NDUFA2	181
LYPLA1	NDUFB6	204
LYPLA1	NDUFC2	229
LYPLA1	NDUFS4	223
LYPLA1	PLB1	869
LYPLA1	SDHD	270
LYPLA1	SLC11A1	226
LYPLA1	SUCLG1	426
LYPLA1	UQCRQ	157
MAN1A1	MGAT1	969
MAN1A1	MGAT4A	556
MAN1A1	ST3GAL1	339
MFSD1	NDUFAB1	170
MFSD1	SLCO2B1	168
MGAT1	MGAT4A	765
MGAT1	ST3GAL1	510
MGAT4A	ST3GAL1	455
NDUFA12	NDUFA2	999
NDUFA12	NDUFA4	996
NDUFA12	NDUFAB1	999
NDUFA12	NDUFAF2	979

NDUFA12	NDUFB5	999
NDUFA12	NDUFB6	999
NDUFA12	NDUFB9	999
NDUFA12	NDUFC2	999
NDUFA12	NDUFS3	999
NDUFA12	NDUFS4	999
NDUFA12	NPC2	196
NDUFA12	SDHD	456
NDUFA12	SUCLG1	488
NDUFA12	UQCRH	987
NDUFA12	UQCRQ	989
NDUFA12	VDAC3	227
NDUFA2	NDUFA4	999
NDUFA2	NDUFAB1	999
NDUFA2	NDUFAF2	958
NDUFA2	NDUFB5	999
NDUFA2	NDUFB6	999
NDUFA2	NDUFB9	999
NDUFA2	NDUFC2	999
NDUFA2	NDUFS3	999
NDUFA2	NDUFS4	999
NDUFA2	PPA2	299
NDUFA2	SDHD	479
NDUFA2	SUCLG1	274
NDUFA2	UQCRH	977
NDUFA2	UQCRQ	997
NDUFA2	VDAC3	150
NDUFA4	NDUFAB1	981
NDUFA4	NDUFAF2	411

NDUFA4	NDUFB5	979
NDUFA4	NDUFB6	981
NDUFA4	NDUFB9	965
NDUFA4	NDUFC2	970
NDUFA4	NDUFS3	968
NDUFA4	NDUFS4	963
NDUFA4	PPA2	406
NDUFA4	SDHD	692
NDUFA4	SUCLG1	414
NDUFA4	UQCRH	890
NDUFA4	UQCRQ	945
NDUFA4	VDAC3	234
NDUFAB1	NDUFAF2	929
NDUFAB1	NDUFB5	999
NDUFAB1	NDUFB6	999
NDUFAB1	NDUFB9	999
NDUFAB1	NDUFC2	999
NDUFAB1	NDUFS3	999
NDUFAB1	NDUFS4	999
NDUFAB1	PPA2	507
NDUFAB1	SDHD	910
NDUFAB1	SUCLG1	503
NDUFAB1	UQCRH	975
NDUFAB1	UQCRQ	989
NDUFAB1	VDAC3	354
NDUFAF2	NDUFB5	941
NDUFAF2	NDUFB6	934
NDUFAF2	NDUFB9	934
NDUFAF2	NDUFC2	944

NDUFAF2	NDUFS3	956
NDUFAF2	NDUFS4	970
NDUFAF2	SDHD	342
NDUFAF2	TYMP	200
NDUFAF2	UQCRH	424
NDUFAF2	UQCRQ	281
NDUFB5	NDUFB6	999
NDUFB5	NDUFB9	999
NDUFB5	NDUFC2	999
NDUFB5	NDUFS3	999
NDUFB5	NDUFS4	999
NDUFB5	PIGF	183
NDUFB5	PPA2	534
NDUFB5	SDHD	933
NDUFB5	SUCLG1	603
NDUFB5	UQCRH	988
NDUFB5	UQCRQ	990
NDUFB5	VDAC3	302
NDUFB6	NDUFB9	999
NDUFB6	NDUFC2	999
NDUFB6	NDUFS3	999
NDUFB6	NDUFS4	999
NDUFB6	PPA2	386
NDUFB6	SDHD	933
NDUFB6	SUCLG1	403
NDUFB6	UQCRH	963
NDUFB6	UQCRQ	975
NDUFB6	VDAC3	305
NDUFB9	NDUFC2	999

NDUFB9	NDUFS3	999
NDUFB9	NDUFS4	999
NDUFB9	PPA2	353
NDUFB9	SDHD	917
NDUFB9	SUCLG1	787
NDUFB9	UQCRH	945
NDUFB9	UQCRQ	995
NDUFB9	VDAC3	193
NDUFC2	NDUFS3	998
NDUFC2	NDUFS4	999
NDUFC2	PPA2	369
NDUFC2	SDHD	812
NDUFC2	SUCLG1	314
NDUFC2	UQCRH	967
NDUFC2	UQCRQ	987
NDUFC2	VDAC3	162
NDUFS3	NDUFS4	999
NDUFS3	PPA2	273
NDUFS3	SDHD	912
NDUFS3	SUCLG1	873
NDUFS3	UQCRH	908
NDUFS3	UQCRQ	985
NDUFS3	VDAC3	375
NDUFS4	PPA2	297
NDUFS4	SDHD	875
NDUFS4	SUCLG1	380
NDUFS4	TYMP	269
NDUFS4	UQCRH	985
NDUFS4	UQCRQ	988

NDUFS4	VDAC3	508
NPC2	SLC7A7	161
NPC2	UQCRH	152
PIGF	TYMP	157
PIK3R5	SUCLG1	294
PIK3R5	TRPM2	155
PLB1	STARD13	173
PLCG2	TBXAS1	169
PPA2	SDHD	221
PPA2	SUCLG1	486
PPA2	UQCRH	250
PPA2	UQCRQ	351
SCN3A	SLC7A7	160
SDHD	SLC11A1	213
SDHD	SUCLG1	975
SDHD	UQCRH	614
SDHD	UQCRQ	739
SDHD	VDAC3	234
SLC11A1	SLC2A5	181
SLC11A1	SLC7A7	315
SLC11A1	SLCO5A1	162
SLC11A1	TBXAS1	192
SLC11A1	TRPM2	903
SLC11A1	TYMP	192
SLC2A5	SLC7A7	246
SLC2A5	SLCO2B1	330
SLC2A5	SUCLG1	178
SLC2A5	TRPM2	900
SLC7A7	SLCO2B1	202

SLC7A7	TBXAS1	161
SLC7A7	TYMP	163
SLCO2B1	SLCO5A1	198
SUCLG1	TYMP	567
SUCLG1	UQCRH	440
SUCLG1	UQCRQ	447
SUCLG1	VDAC3	415
UQCRH	UQCRQ	999
UQCRH	VDAC3	358
UQCRQ	VDAC3	255
